# Serum biomarker-based osteoporosis risk prediction and the systemic effects of *Trifolium pratense* ethanolic extract in a postmenopausal model

**DOI:** 10.1186/s13020-022-00622-7

**Published:** 2022-06-14

**Authors:** Yixian Quah, Jireh Chan Yi-Le, Na-Hye Park, Yuan Yee Lee, Eon-Bee Lee, Seung-Hee Jang, Min-Jeong Kim, Man Hee Rhee, Seung-Jin Lee, Seung-Chun Park

**Affiliations:** 1 College of Veterinary Medicine and Cardiovascular Research Institute, Kyungpook National University, 80 Daehak-ro, Daegu, 41566 Republic of Korea; 2 Centre of IoT and Big Data, Universiti Tunku Abdul Rahman, 31900 Kampar, Perak Malaysia; 3 Laboratory Animal Center, Daegu-Gyeongbuk Medical Innovation Foundation, Daegu, Republic of Korea; 4 Reproductive and Development Toxicology Research Group, Korea Institute of Toxicology, Daejeon, Republic of Korea; 5 Teazen Co. Ltd., Gyegok-myeon, Haenam-gun, Jeollanam-do 59017 Republic of Korea

**Keywords:** Machine learning, Osteoporosis, Postmenopause, Red clover, *Trifolium pretense*, XGBoost

## Abstract

**Background:**

Recent years, a soaring number of marketed *Trifolium pratense* (red clover) extract products have denoted that a rising number of consumers are turning to natural alternatives to manage postmenopausal symptoms. *T. pratense* ethanolic extract (TPEE) showed immense potential for their uses in the treatment of menopause complications including osteoporosis and hormone dependent diseases. Early diagnosis of osteoporosis can increase the chance of efficient treatment and reduce fracture risks. Currently, the most common diagnosis of osteoporosis is performed by using dual-energy x-ray absorptiometry (DXA). However, the major limitation of DXA is that it is inaccessible and expensive in rural areas to be used for primary care inspection. Hence, serum biomarkers can serve as a meaningful and accessible data for osteoporosis diagnosis.

**Methods:**

The present study systematically elucidated the anti-osteoporosis and estrogenic activities of TPEE in ovariectomized (OVX) rats by evaluating the bone microstructure, uterus index, serum and bone biomarkers, and osteoblastic and osteoclastic gene expression. Leverage on a pool of serum biomarkers obtained from this study, recursive feature elimination with a cross-validation method (RFECV) was used to select useful biomarkers for osteoporosis prediction. Then, using the key features extracted, we employed five classification algorithms: extreme gradient boosting (XGBoost), random forest, support vector machine, artificial neural network, and decision tree to predict the bone quality in terms of T-score.

**Results:**

TPEE treatments down-regulated nuclear factor kappa-B ligand, alkaline phosphatase, and up-regulated estrogen receptor β gene expression. Additionally, reduced serum C-terminal telopeptides of type 1 collagen level and improvement in the estrogen dependent characteristics of the uterus on the lining of the lumen were observed in the TPEE intervention group. Among the tested classifiers, XGBoost stood out as the best performing classification model with the highest F1-score and lowest standard deviation.

**Conclusions:**

The present study demonstrates that TPEE treatment showed therapeutic benefits in the prevention of osteoporosis at the transcriptional level and maintained the estrogen dependent characteristics of the uterus. Our study revealed that, in the case of limited number of features, RFECV paired with XGBoost model could serve as a powerful tool to readily evaluate and diagnose postmenopausal osteoporosis.

**Supplementary Information:**

The online version contains supplementary material available at 10.1186/s13020-022-00622-7.

## Background

The world population is ageing as a result of our pursuit of longevity. According to the report released by the United Nation in 2020, the number of populations that is aged 65 years or older is estimated to be 727 million worldwide [1]. Women generally have life expectancy longer than men and because of that, women have accounted for 55% of the global population aged over 65 years old or more. Osteoporotic bone fracture is one of the major health consequences in elderly people [2]. The prevalence of osteoporosis increases in both genders with aging, but more significantly in women over 50 years old [3]. At menopause, estrogen deficiency causes the expedition of bone resorption through several mechanism of actions. Conventional therapies for post-menopausal osteoporosis such as antiresorptive drugs (estrogen, calcitonin, bisphosphonates) and callus formation drugs (parathyroid hormone) have low long-term compliance and side effects; over and above, the cost of treatment can be prohibitive [4, 5]. Consequently, scientists have sought for alternative therapeutics including nutrients and botanicals.

*Trifolium pratense* L. (Fabaceae) (red clover), also known as *hong che zhou cao* or *san ye cao*, has long been used ethnopharmacologically to treat a variety of illnesses, including asthma, whooping cough, and gout [6, 7]. In recent years, it has been promoted and marketed as dietary supplements to help manage menopause symptoms, cholesterol levels, and osteoporosis. There are a total of 1545 commercial red clover products, 78 of which are Canadian Licensed [8]. The soaring number of red clover extract products on the market denoting an increasing number of consumers are seeking natural alternatives to treat postmenopausal symptoms. Scientific studies have been conducted to elucidate the effects of red clover extract and its active ingredients on vasomotor symptoms and bone quality [9, 10]. Its benign effects on the breast, endometrium, and neural structure have also been reported [11].

Previous in vivo studies on the effects of red clover have almost exclusively focused on the macroscopic and mechanical properties of bones [12, 13], there have been relatively few studies on the molecular effects of red clover extract in bones. As a result, our study sought to systematically elucidate the anti-osteoporosis and estrogenic properties of red clover extract on both the macroscopic and molecular levels. Additionally, we sought to validate the HPLC method for determining the active ingredients in this extract in order to ensure the extract’s high quality, which is critical for its safety and efficacy.

The deterioration of bone quality occurs silently until a fracture occurs. Thus, early detection of osteoporosis increases the likelihood of effective treatment and decreases the risk of fracture. Currently, the most frequently used method for diagnosing osteoporosis is dual-energy x-ray absorptiometry (DXA or DEXA). However, DXA's primary limitation is that it is inaccessible and prohibitively expensive in rural and remote areas for primary care inspection. As a result, alternative techniques for early detection of this silent disease is necessary.

Advanced machine learning models (e.g., XGBoost) and feature selection techniques (e.g., Recursive Feature Elimination Cross Validation (RFECV)) in combination with biomarkers may provide a reliable and accessible alternative to DXA for osteoporosis diagnosis. These studies, however, are extremely limited. Several previous studies attempted to predict osteoporosis using machine learning, their studies employed aggregated data such as demographic data and medical records [14, 15]. TK Yoo, SK Kim, DW Kim, JY Choi, WH Lee, E Oh and E-C Park [15] predicted osteoporosis using demographic characteristics (e.g., age, height, and BMI) and medical records (e.g., duration of menopause, history of fracture, and diabetes) from the Korea National Health and Nutrition Examination Surveys.

The current study hypothesizes that biomarkers could be used as input features for osteoporosis prediction. This is because biomarkers have demonstrated a high potential for being a useful, relatively inexpensive, and non-invasive tool for osteoporosis assessment [16]. These biomarkers provide functional information that aids in the early detection and treatment of major diseases such as osteoporosis [17]. The association between osteoporosis and diseases like chronic kidney [18] and/or liver disease [19], diabetes [20–22], obesity [23, 24], and chronic obstructive pulmonary disease [25] has been raised in the previous reports. As a result, serum biomarkers associated with these diseases may be useful in diagnosing osteoporosis.

## Methods

### Preparation of ethanolic extract of *Trifolium pratense* flower

*T. pratense* ethanolic extraction and species identification were performed using the previously described method [26]. *T. pratense* was obtained from a certified company, Teazen Co. Ltd. (Anyang-si, Republic of Korea), and was cultured and originated from Albania (Certificate of Origin Number A19690728 was issued on 2019-02-11 by Industrie- und Handelskammer Würzburg-Schweinfurt, verification code: GSY4-3M2C-CP9C), with the voucher number P-338852. *T. pratense* was collected with the necessary institutional permissions.

This study is in accordance with local and national regulations. DaeHo Co. Ltd. (Gyeonggi-do, Republic of Korea) extracted the product in accordance with Good Manufacturing Practice (GMP) standards and the Food Item Manufacturing Report. Briefly, macerated *T. pratense* leaves and flowers (30 kg) were mixed with extraction solvent (30% ethanol) in a 1:30 ratio. The sample was extracted twice for 3 h at 85 °C, and the extract from each extraction step was filtered through a 1-μm filtration. The resulting *T. pratense* ethanolic extract (TPEE) was then combined with dextrin in a 7:3 ratio (extract:dextrin) during a spray-drying step at 180 °C as a product prototype for animal testing.

### Chemical characterization of *T. pratense* using UPLC-ESI–MS/MS analysis

UPLC was performed with a Waters Acquity UPLC system (Waters Corporation, Milford, USA). The samples were separated on a Waters BEH C_18_ column (2.1 × 150 mm, 1.7 µm) at room temperature. The mobile phase consisted of water (A) and acetonitrile (B), both acidified with 0.1% formic acid. The elution gradient was set as follows: 0–1 min, 5% B; 1–20 min, 5–70% B; 20–24 min, 70–100% B; 24–27 min, 100% B; 27–27.1 min, 100–5% B; 27.1–30 min, 5% B. The flow rate was 0.4 mL/min and the sample loading volume was 1 µl. The UPLC was coupled to an LTQ-Orbitrap XL hybrid mass spectrometer (Thermo Electron, Bremen, Germany) via an ESI interface. The samples were analyzed in positive ion mode and the conditions of the ESI source were the same as previously used [27]. The representative chromatogram and spectra were shown in Additional file 1. HPLC quantification of the indicative compounds, biochanin A (BCA) and formononetin (FMT) in TPEE was carried out and reported in our previous study [28].

### Validation of analytical method

The specificity of the method was ascertained by analyzing the standard compounds and the extract. The peaks for BCA and FMT in the sample were confirmed by comparing the retention times of the sample peak with that of the standard. The peak purity of those compounds was assessed by comparing the spectra at two levels, viz; peak start and peak end positions.

The accuracy of an analytical method is the extent to which test results generated by the method and the true value of analytes. The precision of a method is the extent to which the individual test results of multiple injections of a series of standards agree. The known concentrations of BCA and FMT (0.015625, 0.03125, 0.0625 and 0.125 mg) were spiked into the extract and analyzed for 5 times for the determination of accuracy and precision of the analytical method.

The lower limit of detection (LOD) is defined as the lowest concentration of analyte in a sample that can be detected, but not necessarily quantitated, under the stated experimental conditions. It can be calculated from the standard deviation of the response and the slope associated with the calibration curve according to the equation: LOD = (SD × 3.3)/slope. The lower limit of quantification (LOQ) is defined as the lowest concentration of analyte in a sample that can be quantifiable under the stated experimental conditions. It can be calculated from the standard deviation of the response and the slope associated with the calibration curve, according to the following equation: LOQ = (SD × 10)/slope.

The linearity of an analytical method is its ability to elicit test results that are directly proportional to the concentration of analytes in samples within a given range. Linearity is determined by a series of 3 injections of 5 standards having 0.0078125–0.125 mg/mL of BCA and FMT. The linear regression analysis was carried out by plotting the peak areas (y-axis) of each compound against the respective concentrations (x-axis) of BCA and FMT. The linearity for the relationship between peak area and concentration was demonstrated by a correlation coefficient (r^2^) greater than 0.99.

### Animal grouping and treatments

Healthy 8-week-old female Sprague–Dawley rats (body weight 190–230 g) were housed in an air-conditioned environment (22 ± 2 °C), with a 12-h light/dark cycle. The animals were given free access to Teklad-certified irradiated global 18% protein rodent diet (2918C, Envigo, USA), which was supplied by Koatech (Gyeonggi-do, Republic of Korea), as well as distilled water. Animal handling followed ARRIVE guidelines and was conducted in accordance with the National Institutes of Health's Guide for the Care and Use of Laboratory Animals. The institutional animal care and use committee at Kyungpook National University, Republic of Korea, approved this study (approval number: KNU 2018–121). The acclimatized rats were either Sham operated (n = 10) or ovariectomized bilaterally (OVX, n = 60). Normal group (n = 10) was solely used for machine learning analysis as positive control.

The OVX rats were divided into six groups at random, with each group consisting of 10 rats. The members of the group were given names based on the treatment they received: Negative control (NC) = OVX + 30% dextrin; Pomegranate extract (PomE) = OVX + 500 mg/kg/day pomegranate extract; Estradiol (E) = OVX + 25 μg/kg/day estradiol; T125 = OVX + 125 mg/kg/day TPEE; T250 = OVX + 250 mg/kg/day TPEE; T500 = OVX + 500 mg/kg/day TPEE. The conversion of TPEE doses and its functional indicator components (FMT and BCA) in rats to human equivalent doses (HED) was illustrated in Additional file 2. One week after the surgery, the treatment began. The same volume of 30 percent dextrin was given to the sham and NC groups via oral gavage. In this study, three positive controls were used: Sham (representing endogenous estradiol in the body), E (administration of estradiol, a positive control drug used for hormone replacement therapy for the treatment of postmenopausal osteoporosis [29]), and PomE (pomegranate extract as a plant extract positive control for osteoporosis intervention [30, 31]). PomE was purchased from Hanil PFC Co., Ltd. (Seoul, Republic of Korea), which is a recognized health functional food by the MFDS (Ministry of Food and Drug Safety). The PomE was previously characterized chemically using LC/MS/MS [32].

At the end of the treatment, the rats were anesthetized with CO_2_ inhalation until unconscious and blood samples were taken via cardiac exsanguination and centrifuged for 10 min at 1,000 × *g* to obtain serum and stored at − 80 °C until used.

### Preparation of specimens

Femurs and uterus for histology examination were immediately fixed in 10% buffered formalin solution. Paraffin embedded blocks (5 μm) were stained with hematoxylin and eosin (H&E) for histology analysis under an optical microscope. While organs used for gene expression and biomarkers analysis were kept at -80 °C until the tests were performed on thawed samples. The cortical bone thickness was analyzed from histology images and measured using Digimizer (MedCalc Software Ltd, Belgium).

### Bone mineral density (BMD) and Micro-CT analysis

The BMD of right femur in each group (n = 6) were measured using micro-CT (Quantum FX micro-CT, Perkin Elmer, USA) using following settings: 90 kV, 180 µA, 10 mm Field of View (FOV). The femur images were reconstructed using Analyze 12.0 software (PerkinElmer, USA). The bone radiomorphometric parameters including BMD (mg/cc), percentage of bone volume/total volume (BV/TV, %), trabecular thickness (Tb.Th, mm), trabecular spacing (Tb.Sp, mm), trabecular number (Tb.N, mm^−1^) were also determined.

### Reverse transcription (RT)- polymerase chain reaction (PCR)

Femur was excised and cleaned of all muscles and connective tissue was removed. The bone was frozen in liquid nitrogen and crushed with pestle and mortar. Total RNA was isolated using guanidinium thiocyanate-phenol–chloroform extraction (TRIzol). TRIzol was added to the bone sample and homogenizer was used in homogenization step. The RNA pellets were dissolved in DEPC water and quantified using a nanophotometer.

### PCR amplification

For expression of the selected genes: receptor activator of nuclear factor-kappa B ligand (RANKL), osteoprotegerin (OPG), osteocalcin (OCN), collagen type 1 (α) (ColA) and alkaline phosphatase (ALP), estrogen receptor (ER α and β), 10 pmol forward primers and reverse primers of each gene was prepared. GAPDH was used as housekeeping gene. Amplification was performed as follows: 40 cycles at 94 °C for 30 s, annealing at the 60 °C for 30 s, and 72 °C for 30 s. The first cycle was conducted at 94 °C for 5 min and the final cycle at 72 °C for 7 min and then ended at 4 °C. The sequences of the primers for each target genes were shown in Additional file 3.

### Biomarker analysis

The serum used for biomarker analysis was kept at -80 °C until the respective tests were performed. Serum biomarker concentrations were determined using an enzyme-linked immunosorbent assay (ELISA) kit (Cusabio Biotech, Wuhan, China). According to the manufacturer's instructions, sample analysis and calibration curves were plotted. Serum biochemistry analysis was done by Medivalley Daegu-Gyeongbuk Medical Cluster.

### Dataset description

A total of 16 biomarkers (8 primary biomarkers and 8 secondary biomarkers) were included in this study as input features to predict bone quality of the test subjects. Primary biomarkers were the biomarkers that are normally known for having direct relationship with bone quality, or commonly known bone biomarkers supported by literatures. Secondary biomarkers were the biomarkers that have indirect impacts towards bone quality. The biomarkers included are listed in Additional file 4.

### Models descriptions

The 5 models used in this study to map the relationship between the 16 biomarkers and osteoporosis. The 5 models are XGBoost, Random Forest (RF), Artificial Neural Network (ANN), Decision Tree (DT), and Support-Vector Machine (SVM). These 5 models are examined because they are accessible and are commonly used in various studies related to osteoporosis [33–35] or biomarkers [36, 37].

DT, RF, and XGBoost are tree-based algorithms with DT being the base unit. DT is an algorithm that splits observations into subsets to optimizes the classification loss function, in this case, it is the Gini Index Function given by:

1 $$\text{Loss }=\sum \left\{{p}_{k}*\left(1-{p}_{k}\right)\right\}$$

where p_k_ is proportion of instances of class k in a particular node.

**Fig. 1:**
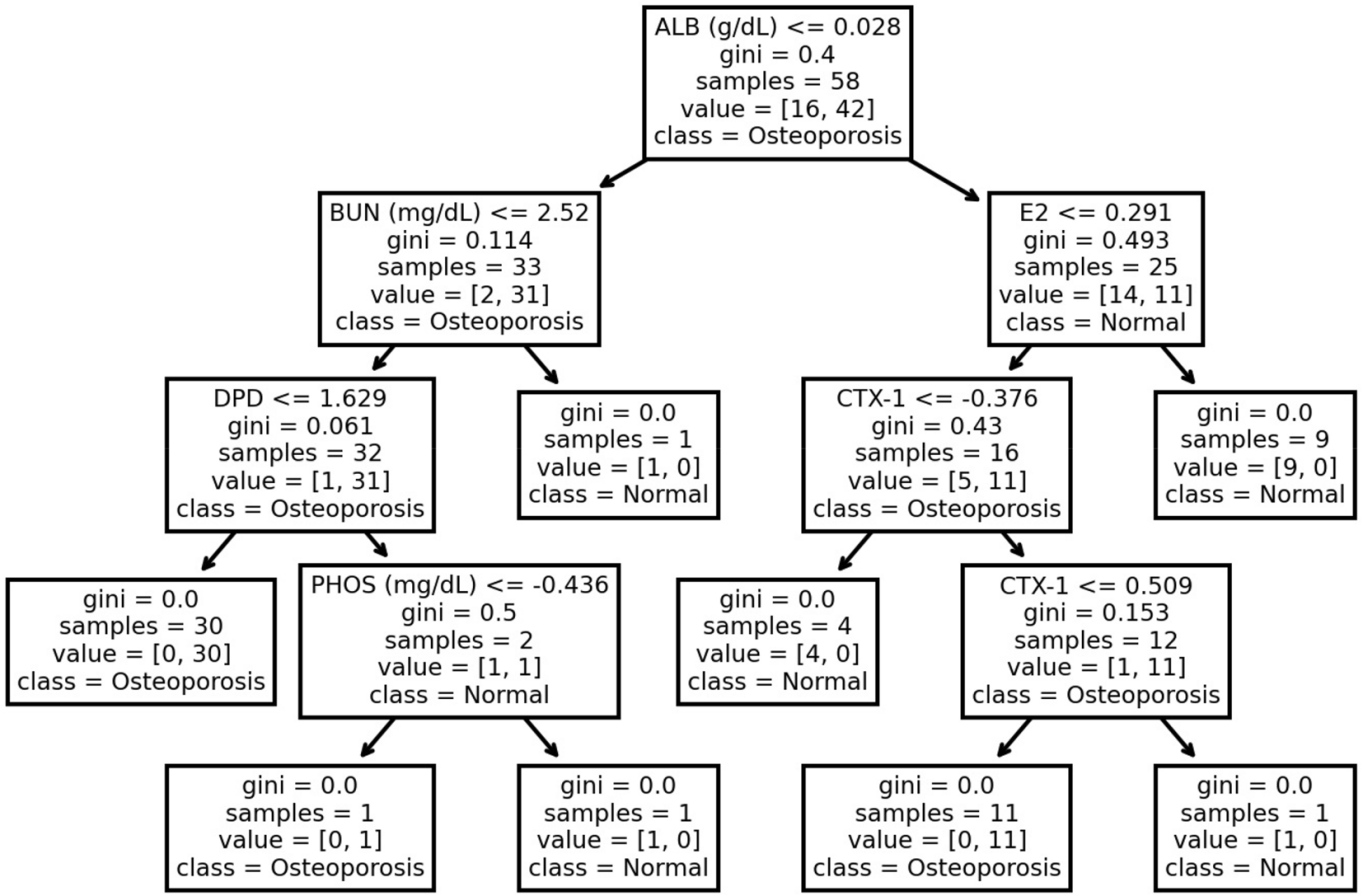
A typical optimized classification decision tree to map the relationship between serum biomarkers and osteoporosis

The DT is commonly used for its interpretability. A possible example of the optimized DT specifically could be visualized in Fig. 1.

RF is a decision-tree-based ensemble machine learning algorithm where a number of decision trees are each fitted to different subsamples. The subsamples are drawn via bootstrapping. The final prediction of a RF is the average prediction of the fitted decision trees. These features reduce tendency for overfitting the data and improve overall prediction performance. Similar to RF, XGBoost is also a decision-tree-based ensemble machine learning algorithm but is optimized via gradient descend (also known as Gradient Boosting). Gradient Boosting optimizes the model by minimizing the loss function such as the binary cross-entropy (CE) loss function:

2 $$\text{CE Loss }= \frac{-1}{N}\sum_{i=1}^{N}{y}_{i}*\text{log}\left(p\left({y}_{i}\right)\right)+\left(1-{y}_{i}\right)*\text{log}(1-p\left({y}_{i}\right))$$

**Fig. 2:**
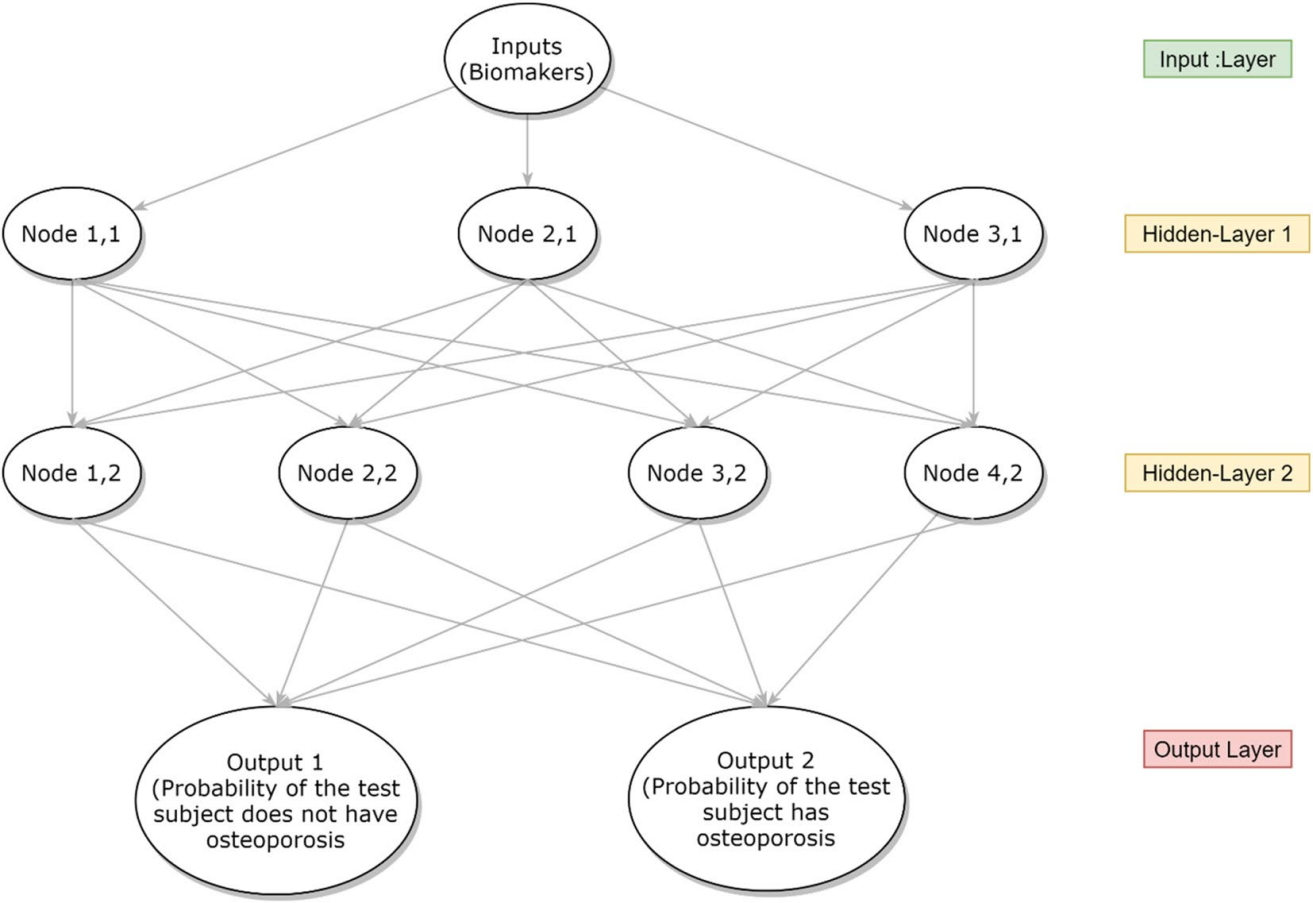
A typical illustration of a neural network to map the relationship between serum biomarkers and osteoporosis

ANN is a series of nodes comprises a linear regression and an activation function to introduce non-linearity (Fig. 2). The ANN is also optimized via gradient descend on the loss function such as the CE loss defined in Eq. (2).

SVM is a machine learning model that defines a decision boundary for classification. The decision boundary can become non-linear by adding the kernel function such as the radial basis function kernel defined below:

3 $$K\left(x, {x}^{^{\prime}}\right)=exp\left( - \frac{|| x-{x}^{^{\prime}}|{\left.\right|}^{2}}{2{\alpha }^{2}}\right)$$

where x and x’ are 2 samples, α is an arbitrary parameter, and $|| x-{x}^{^{\prime}}|{\left.\right|}^{2}$ is the Euclidean distance between x and x’.

### Feature selection with recursive feature elimination with cross validation (RFECV)

Firstly, the input features are normalized via Standard Scaler:

4 $${X}_{norm}=\frac{x- \overline{x}}{\sigma }$$

Next, the input features undergone feature selection and are ranked based on their importance defined by a widely adopted technique known as Recursive Feature Elimination with Cross Validation (RFECV) as large number of input features does not always contribute to better prediction performance. RFECV is used in this study because RFECV has shown to evaluate a model’s generalization ability with small datasets well. Furthermore, RFECV is widely adopted for similar tasks in biomedical fields such as gene selection and cancer diagnosis [38, 39].

The parameters used for RFECV are as below:

- Number of input variables per feature set = 10
- Classifier Model = Random Forest
- K-hold subsets = 5
- Scoring Metrics = F1-Score

The F1-score is the geometric mean of recall and precision as is defined as Eq. (5). F1-score was used instead of accuracy due to dataset imbalance explained in the ‘Prediction tools and model performance metrics’ section.

5 $$\text{F}1 = \frac{2}{\frac{1}{recall}+ \frac{1}{precision.}}$$

The feature selection process was repeated 50 times to obtain stable result due to the stochastic nature of RFECV. Each biomarker was scored, ranked, and aggregated via median over the 50 iterations. F1-score is used as the scoring metrics due to imbalance sample distribution where large majority of samples have osteoporosis. Biomarkers that are deemed relevant in predicting osteoporosis in the test subjects will received rank of 1, whereas biomarkers that are not deemed relevant in predicting osteoporosis in the test subjects will receive rank of 2 onwards, representing the relevancy in descending order.

### Prediction tools and model performance metrics

The selected biomarkers (biomarkers with rank 1) were then served as input variables to the machine learning models. The machine learning models were trained to learn the association between the selected biomarkers and the severity of osteoporosis. According to the National Osteoporosis Foundation, the severity of osteoporosis is measured by T-score of Bone Mass Density (BMD) and is classified as shown in Additional file 5.

$$\text{where} \; \text{T-Score}= \frac{\text{BMD} - \text{Average} \;\text{ BMD} \;\text{among} \;\text{Healthy} \;\text{Young} \;\text{Adults}}{\text{Standard} \;\text{Deviation} \;\text{BMD} \;\text{among} \;\text{Young} \;\text{Adults}}$$

Therefore, the models will be trained to classify the osteoporosis severity of the test subjects. Since there are no defined standard of osteoporosis severity for non-human test subjects, the mean and standard deviation used for the T-Score will be derived from test subjects from the positive control (Sham and normal groups) in this study.

After eliminating the samples that contained missing data, 73 samples were used in this study and the distribution of samples over the 3 severity groups were shown in Additional file 6.

Due to the limitation in number of samples from the osteopenia severity group, the osteopenia severity group was re-labelled as ‘No Osteoporosis’. Hence, only 2 classes remained instead of 3. Five different machine learning models were considered, and their respective performances are compared. The 5 machine learning models include XGBoost, RF, SVM, ANN, and DT.

The models were trained on 58 samples (80% training set) and evaluated on the other 15 unobserved samples (20% test set). The performance of the model was evaluated based on the classification F1-score on the test set. F1-score was selected due to imbalance sample distribution where large majority of test subjects have osteoporosis. Similar to the feature selection process, the assessment of the machine learning models was also evaluated 50 times to obtain stable results. The training samples and test samples were shuffled in every iteration and the results (F1-score) were aggregated via mean and standard deviation. Additional file 7 shows the general modelling framework for the prediction model.

### Statistical analysis

The data were expressed as mean ± standard deviation (SD), and the statistical significance (*p* < 0.05) was determined by two-way analysis of variance (ANOVA) with Tukey's post-hoc analysis (GraphPad Prism 5.01, La Jolla, CA, USA).

## Results

### Chemical characterization of TPEE and validation of analytical method

#### Identification of chemical constituents in TPEE using UPLC-ESI–MS/MS analysis

Constituents in TPEE identified are daidzein (DZN), genistein (GNT), formononetin (FMT), and biochanin A (BCA) with different retention time, 8.61, 10.22, 11.91, and 14.00 min (Additional file 1). The mass spectra for each compound were also shown.

#### Specificity

Specificity of the analytical method ensures that the signals measured come from the indicative compounds (BCA and FMT) in TPEE and there is no interference from diluents, extract materials and mobile phase. Photodiode array detection also supported the specificity of the method and provided evidence for the homogeneity of the peaks of analytes (Additional file 8). Peaks obtained from recovery experiments were checked for uniformity using UV spectra taken from different points of the peak of interest. These spectra were superimposed whenever overlaid; showing that there were no other co-eluting peaks, in every instance for each of the analytes. The data obtained in the validation study proved that the proposed method is validated and can be utilize for the determination and quantification of BCA and FMT.

#### Accuracy and precision

The accuracy, intra-day precision (repeatability), and inter-laboratory precision (reproducibility) of this assay method were shown. The accuracy of the analysis method was measured as the recovery of analytes (Additional file 9). The recovery range for BCA and FMT were 84.58–91.27% and 99.63–106.36%, respectively, which fell in the acceptable range of 80–120%. The precision was measured in terms of repeatability and reproducibility (Additional file 9) of this assay method was within the limit for all tested concentration according to the guidelines for analytical method development and validation [40, 41]. The percent relative standard deviation (%RSD) is within the limits (%RSD < 2%), which indicate that, the assay method is validated depending on the precision.

#### LOD and LOQ

The method sensitivity has been checked practically where experimental LODs were 0.00639 mg/mL and 0.00519 mg/mL for BCA and FMT, respectively. The LOQ of BCA was 0.01936 mg/mL, and FMT was 0.01573 mg/mL (Additional file 10).

#### Linearity

The analytical calibration curves were constructed for BCA and FMT. The linear regression equation for BCA is y = 7265.0x + 109.44. The linear regression equation for FMT is y = 5002.2x + 58.611. The correlation coefficients (r^2^) of BCA and FMT were 0.9999 (Additional file 11).

### Effects of TPEE on the uterus of OVX rats

**Fig. 3:**
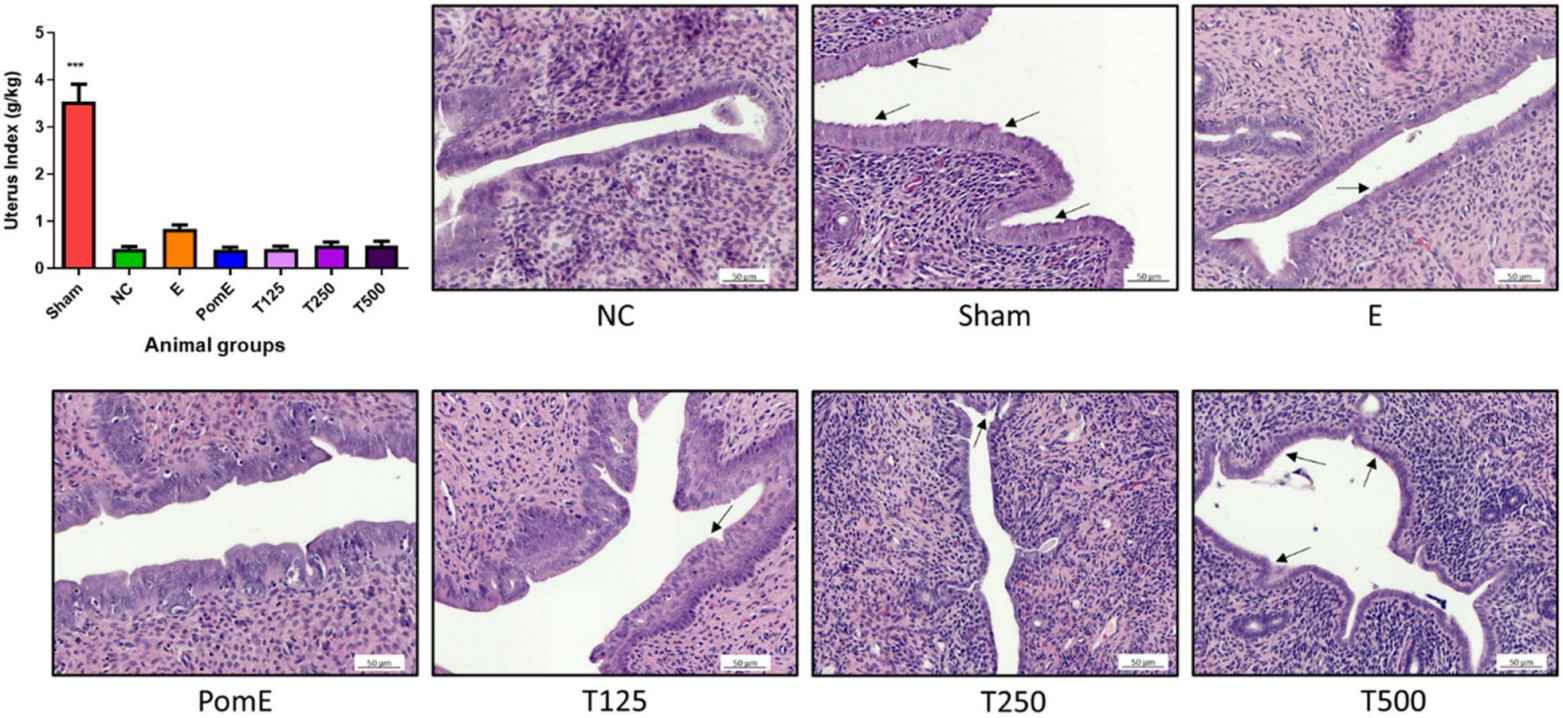
The effects of TPEE (T125, T250 and T500) or positive controls (Sham, PomE and E) on uterus index in OVX rats. Data were presented as mean ± SD, n = 6. ***p < 0.001 compared with NC group determined by two-way ANOVA test. Representative images of H&E staining of the rats’ uterus. The arrows indicated the ciliated cells on the linings of the epithelium cells. All pictures are stained with H&E and examined under × 400 magnification. Scale bar, 50 µm

The uterus of the Sham rats is larger and thicker than that of the OVX rats. The uterus index was calculated as: $\text{Uterus} \;\text{index} =\frac{\text{Uterus} \;(\text{g})}{\text{Body} \;\text{weight} \;(\text{kg})}$. As illustrated in Fig. 3, the Sham group's uterus index was approximately 7.3-fold that of the other treated OVX groups. TPEE shows no significant effect on the uterus index compared to NC. Sham's uterine glands have been found to be slightly elongated. Notably, the uterine lumen of the Sham group was lined by a stratified columnar epithelium with ciliated cells interspersed (Fig. 3). On the other hand, the NC group lacked ciliated cells on the lumen lining of the uterus, resulting in a smooth surface of the lumen. In contrast to NC, ciliated cells were observed in the uterine lumen lining of the T500 group, which is similar to the Sham group, as evidenced by a rough surface on the lumen epithelium.

### Effects of TPEE on trabecular and cortical bone thickness in the femur of OVX rats

**Fig. 4:**
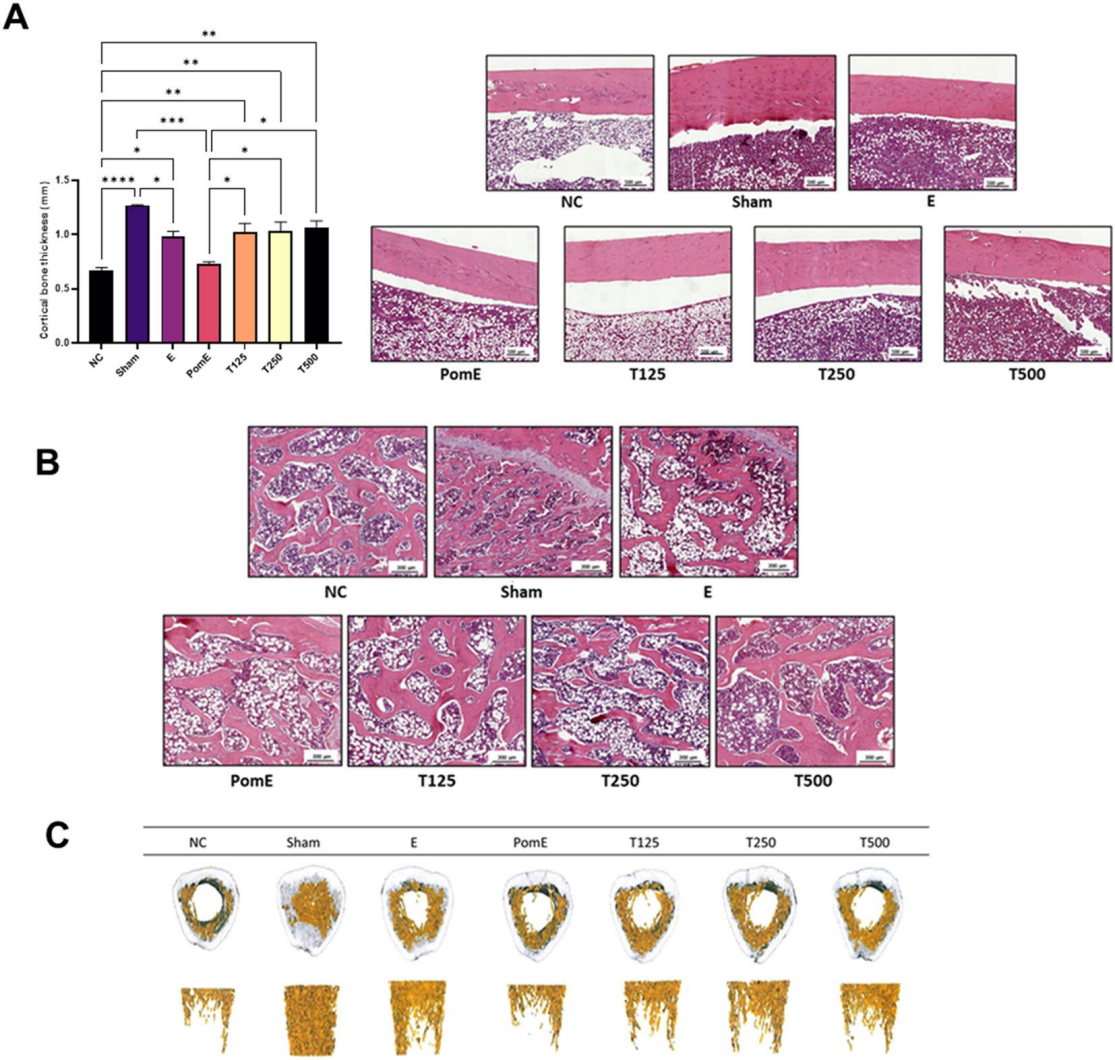
The effects of TPEE on cortical and trabecular bones. **A** The cortical bone thickness and the representative images of H&E staining of rats’ femur. All pictures are stained with H&E and examined under × 2.2 magnification. Scale bar, 500 µm. Data were presented as mean ± SD, n = 6. *p < 0.05; **p < 0.01; ***p < 0.001; ****p < 0.0001 determined by two-way ANOVA test. **B** Trabecular bone tissue was stained with H&E and examined under × 400 magnification. Scale bar, 200 µm. **C** The representative images of the 3D architecture of trabecular bone analyzed by microCT analysis

Figure 4C shows representative micro-CT 3D reconstruction images of the femurs. Table 1 summarizes the values of BMD (mg/cc), BV/TV (%), Tb.Th (mm), Tb.Sp (mm), and Tb.N (mm^−1^) for all the groups. NC group had a significant decrease in BMD (366.33 ± 32.07 mg/cc), BV/TV percentage (1.58 ± 0.45%), Tb.Sp (0.09 ± 0.01 mm), and Tb.N (1.58 ± 0.06 mm^−1^). The groups treated with TPEE did not show significant improvement in the microstructure parameters. In all groups, there was no significant change in BV/TV, Tb.Sp, or Tb.N in the femurs. As shown in Fig. 4C, the OVX groups experienced a significant loss of trabecular bone volume (NC, PomE and all the TPEE groups), while estradiol administration (E group) restored the trabecular bone volume. However, statistical analysis revealed that the E group's BV/TV was not statistically different from that of the NC group (Table 1).

**Table 1:** Effect of red clover extract on the microstructure and BMD of the OVX rats

Parameters	NC	Sham	E	PomE	T125	T250	T500
BMD (mg/cc)	366.33 ± 32.07	723.45 ± 53.81 ^a^	433.33 ± 31.81 ^a^	398.71 ± 17.35	404.95 ± 20.44	405.64 ± 2.34	406.59 ± 9.51
BV/TV (%)	1.58 ± 0.45	13.11 ± 2.24 ^a^	2.51 ± 0.82	1.51 ± 0.66	2.78 ± 0.51	2.75 ± 0.84	1.98 ± 0.42
Tb.Th. (mm)	0.50 ± 0.03	0.47 ± 0.03	0.57 ± 0.03 ^a^	0.53 ± 0.03	0.54 ± 0.05	0.59 ± 0.02 ^a^	0.54 ± 0.04
Tb.Sp (mm)	0.09 ± 0.01	0.11 ± 0.01 ^a^	0.08 ± 0.02	0.10 ± 0.01	0.08 ± 0.01	0.10 ± 0.02	0.09 ± 0.02
Tb.N (mm^−1^)	1.58 ± 0.06	1.81 ± 0.08 ^a^	1.62 ± 0.10	1.61 ± 0.10	1.48 ± 0.02	1.51 ± 0.03	1.63 ± 0.09

Results are presented as mean ± S.D

^a^Indicate that the mean differs significantly from NC group based on one-way ANOVA, followed by Tukey’s post hoc test

In comparison to NC, the TPEE-treated groups had a thicker cortical bone structure, but lacked dose-dependent effect (Fig. 4A). The bone trabeculae structure exhibited distinct disorder, loosening, and breakage in the NC group, while Sham group exhibited compact trabeculae structure. T250 and T500 groups showed increased trabecular space which is similar to that of NC (Fig. 4B). Increases in the marrow cavities were observed in the NC and T125 groups.

### Regulatory effects of TPEE on RANKL and ER β gene expression in the bone

**Fig. 5:**
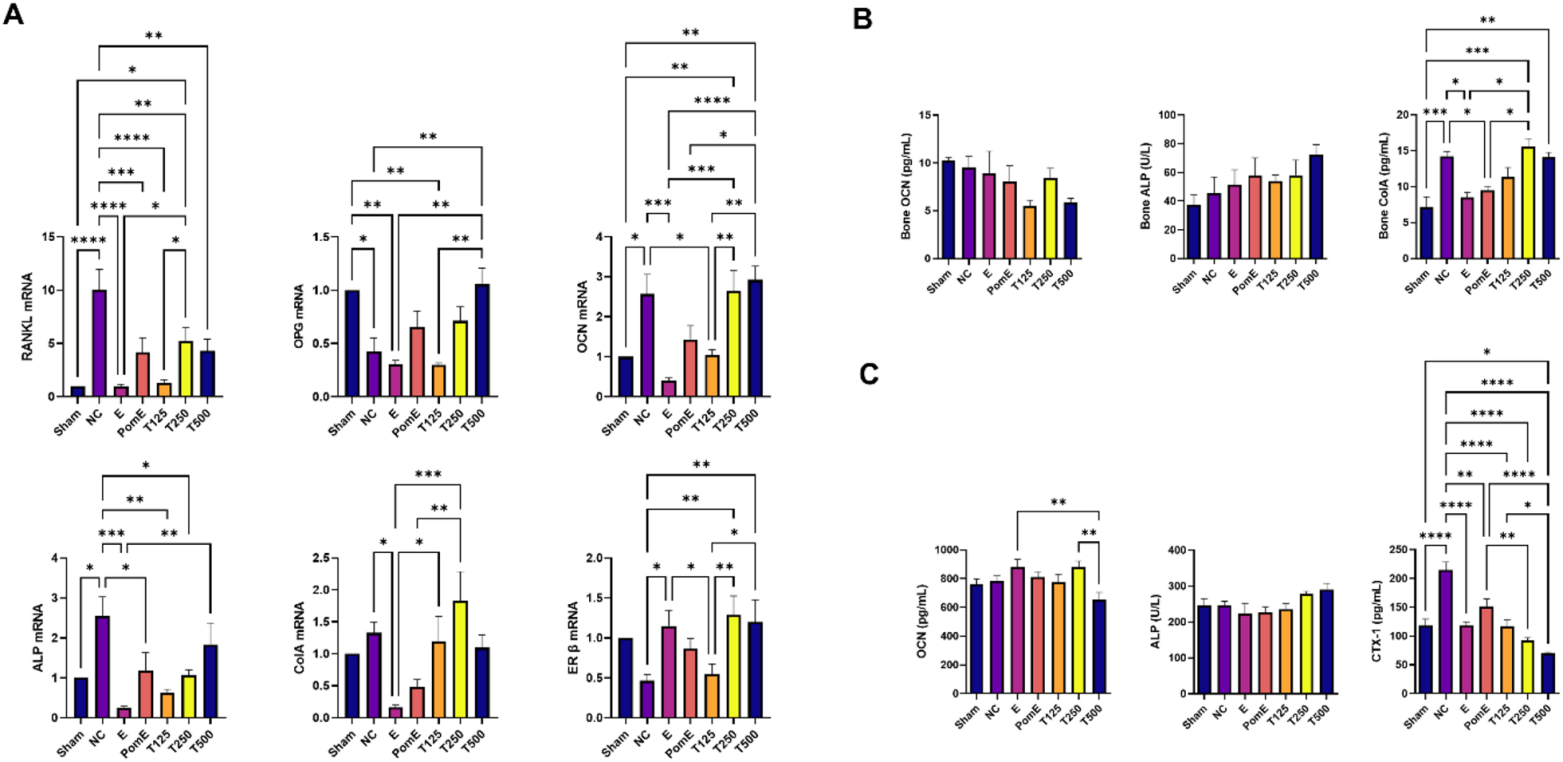
Real-time PCR analysis for mRNA expression of RANKL, OPG, OCN, ALP, ColA, and ERβ in the tibia (**A**). The levels of osteoblastic and osteoclastic biomarkers in OVX rats’ tibia (**B**) and serum (**C**). Data are expressed as mean ± SD (n = 6). Data were presented as mean ± SD, n = 6. *p < 0.05; **p < 0.01; ***p < 0.001; ****p < 0.0001 determined by two-way ANOVA test, followed by Tukey’s post hoc test

At the transcriptional level, the expression of RANKL was down-regulated by TPEE treatment (Fig. 5A). However, TPEE treatment did not show significant effects on other osteoblastic genes such as OPG, OCN, ALP and ColA. TPEE up-regulated ER β gene expression in the tibia but not ERα gene (data not shown) for T250 and T500 groups. The levels of ER protein in the tibia of OVX rats were determined by ELISA, demonstrating that TPEE increased ER protein expression in the tibia (Additional file 12).

### Effects of TPEE on bone and serum biomarker levels

The levels of tibial ALP, OCN, and ColA proteins in the TPEE-treated groups did not differ significantly from the NC group (Fig. 5B). Among the groups, the T500 group showed the greatest improvement in phosphorus content. In this study, serum creatinine (CRE) and blood urea nitrogen (BUN) levels showed no significant increase in the treated groups when compared to the NC and Sham groups (Additional file 13). TPEE treatment reduced serum C-telopeptide of type I collagen (CTX-1) levels in OVX rats in a dose-dependent manner (T125 = 116.7 ± 27.8 pg/mL, T250 = 92.3 ± 11.7 pg/mL, T500 = 69.6 ± 6.4 pg/mL) (Fig. 5C). The serum OCN levels in the treated groups were not statistically different from those in the NC and Sham groups. The serum level of estradiol was significantly higher in all treated groups than in the NC group, but did not differ significantly from the Sham and E groups.

### Recursive Feature Elimination with a Cross-Validation (RFECV) ranking for each serum biomarkers

**Fig. 6:**
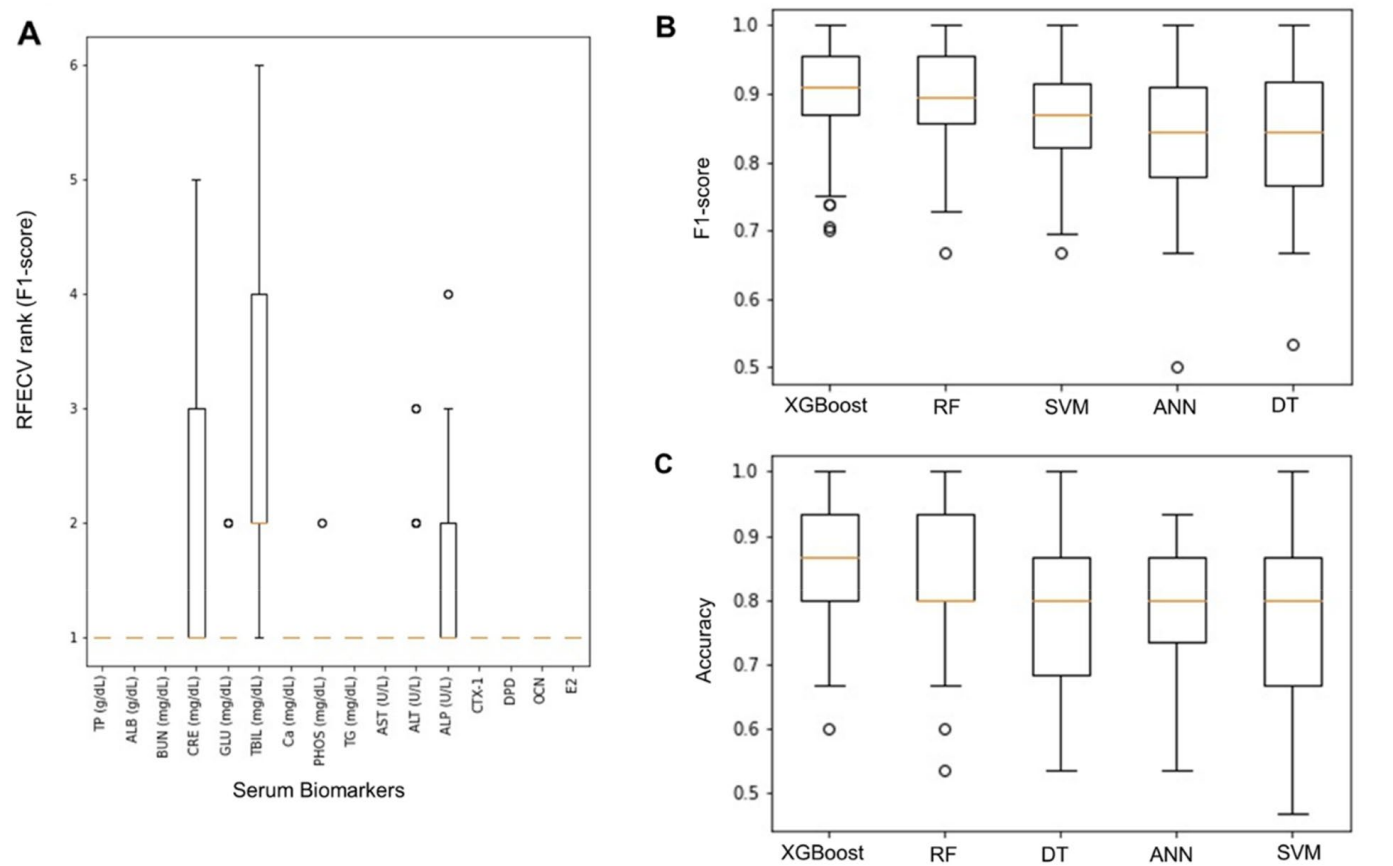
Recursive Feature Elimination with a Cross-Validation (RFECV) ranking for each serum biomarkers based on F1-score (**A**). Boxplot of each model performance **B** F1-score and **C** accuracy over 50 iterations

After normalization, the input features undergone feature selection process via RFECV. The selection process is repeated 50 times to ensure stability in results. According to the RFECV ranking, all biomarkers received a median ranking of 1 except TBIL (Fig. 6A). Although the ranks for CRE, TBIL, and ALP fluctuated throughout the 50 repeated iterations of the feature selection process, the median aggregate ranks for both CRE and ALP were 1. Therefore, only TBIL was eliminated from the input features.

**Fig. 7:**
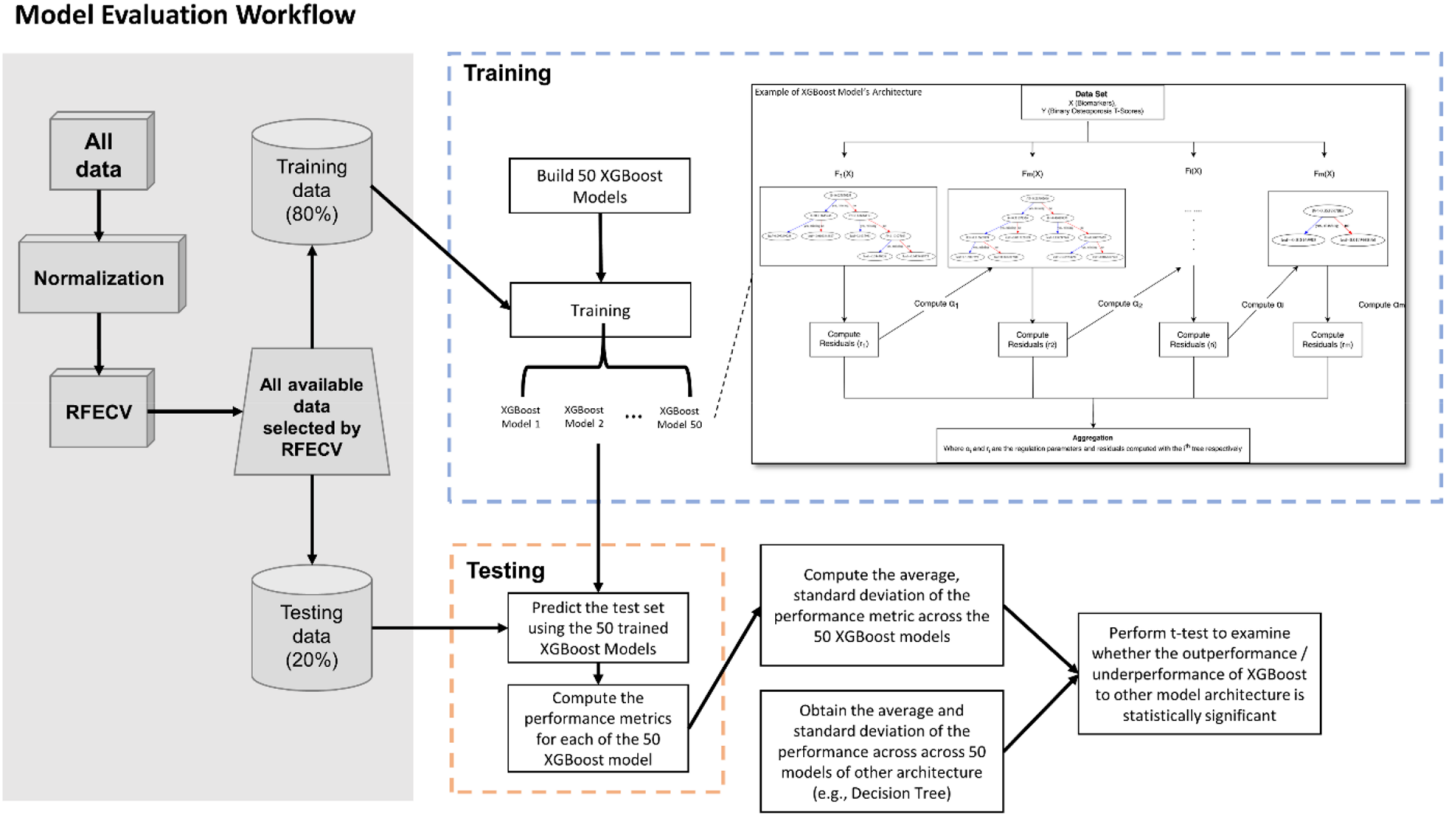
A schematic of the XGBoost model evaluation workflow. The shaded area indicates the data pre-processing (including normalization and feature selection) and data partitioning. The boxes within the dashed lines represents training and testing procedures where F_i_(X) denoting the tree function, where i denoting the ith tree

### Evaluation of machine learning models’ prediction performance

Five machine learning models were trained on the selected input features to predict the osteoporosis severity group of the test subjects. Each model was trained and evaluated 50 times to ensure reliable results. The performance of the models over the 50 iterations in descending order are shown in Table 2. The results from Table 2 shows that the selected biomarkers showed generally satisfactory performance in predicting the osteoporosis severity of the test subjects.

**Table 2:** Prediction performance (F1-score and accuracy) of the different machine learning models over 50 iterations

Models	F1-Score		Accuracy	
	Average	Standard Deviation	Average	Standard Deviation
XGBoost	90.54%	7.21%	86.00%	8.67%
Random Forest	89.15%	7.80%	83.00%	10.76%
Support Vector Machine	85.20%	7.41%	77.47%	12.07%
Artificial Neural Network	83.39%	9.47%	78.13%	10.75%
Decision Tree	83.35%	9.29%	78.40%	10.55%

Figure 6B and 6C show that XGBoost stood out as the best performing classification model based on the performance metrics. It has the highest average F1-score and average accuracy while retaining minimal standard deviation of F1-score and standard deviation of accuracy over the 50 iterations compared to the other 4 models. The performance of RF model was very close to that of XGBoost but having slightly lower average F1-score and accuracy and higher standard deviation of F1-score and accuracy. SVM, ANN, and DT fell behind in both average and standard deviation of F1-score and accuracy over the 50 iterations. Table 3 represents statistical significance (p-value) of the difference of performance (accuracy and F1-score) between XGBoost and the other models. Each model is trained and tested 50 times to obtain 50 accuracy score and 50 F1-score. Firstly, t-test is conducted between 2 models’ 50 accuracy scores to obtain the respective p-value. Then, t-test is conducted again between 2 models’ 50 F1-score to obtain the respective p-value. It is evident that XGBoost is the best performing model. In addition, difference in performance between XGBoost and other models is statistically significant, with RF being the exception (Fig. 7).

**Table 3:** Statistical significance (p-value) of the difference of performance (accuracy and F1-score) obtained from the t-test conducted between XGBoost and other models

Models	P-value	
	F1-Score	Accuracy
XGBoost < > SVM	< 0.01	0.011
XGBoost < > DT	0.016	0.065
XGBoost < > ANN	< 0.01	< 0.01
XGBoost < > RF	0.56	0.43

## Discussion

BCA and FMT are the potential active ingredients in TPEE and they were commonly known as the indicative compounds in red clover extract [28, 42, 43], therefore BCA and FMT in the sample were confirmed using HPLC analytical method. In this study, TPEE was prepared in accordance with GMP standards and the Food Item Manufacturing Report in this study. Analytical validation has fundamental importance in the scope of GMP for herbal products, therefore validation of HPLC analytical method was performed. Validation is the process of confirming that a method satisfies the requirements for a particular use or application by providing objective evidence. Accurate validation of analytical methods is required to ensure the high quality of products, which has a direct impact on their safety.

TPEE treatment improved estrogen-dependent uterine characteristics such as ciliated cells on the lumen lining. Our results indicated that, despite the fact that estradiol was administered to OVX rats (E group), its effect was not comparable to that of the Sham group or significantly different from that of the other treated OVX groups. This result further demonstrated that endogenous estrogen was more effective than exogenous estrogen at maintaining uterine size in OVX rats and postmenopausal women. TPEE did not have a significant effect on the uterus index in OVX rats at the tested concentration.

Trabecular microstructure and BV/TV critical indicators of bone fracture and strength [44]. It should come as no surprise that the Sham group had the highest BV/TV ratio in our study. In comparison to the Sham group, the OVX groups demonstrated a significant decrease in BMD, BV/TV, Tb.Th, Tb.Sp, and Tb.N. Our findings indicated that orally administered estradiol and phytoestrogens had no effect on the bone microstructure over the course of treatment. According to Yen, Qi [45], who conducted a comparison of the therapeutic efficacy of estrogen and parathyroid hormone (PTH), PTH outperformed estrogen as a potent stimulator of bone formation and has the ability to restore lost cancellous bone in osteopenic OVX rats. TPEE treatment had no effect on femoral BMD or other microstructure parameters in the OVX rats. This lack of significant change in femoral BMD is consistent with a previous study in which menopausal women were treated for 12 weeks with red clover extract [46].

Histology analysis of trabecular bone demonstrated that bone marrow cellularity increased dose dependently in OVX rats treated by TPEE. NC group displayed reduction in bone marrow cellularity compared to Sham group. Notably, the reduction of bone marrow cellularity was found with a corresponding increase in bone marrow adiposity [47]. The increase of adipocyte disrupts the micro-environmental equilibrium essential to maintain osteogenesis [48]. According to Verma and colleagues [47], increased adipose tissue is associated with decreased bone formation due to a shift in stromal cell differentiation from osteoblastic to adipocytotic pathways. TPEE treatment may inhibit stromal cell differentiation into adipocytic cells. Thus, TPEE-treated OVX rats had significantly lower serum TG levels as reported in our previous study [26].

RANK is a receptor located on surface osteoclasts, both precursor and mature. RANKL and OPG are the ligands that binds to the RANK receptor, these ligands are synthesized and secreted primarily by osteoblasts and bone marrow stromal cells [49]. When RANK is activated by the RANKL, osteoclast differentiation is initiated, and bone resorption is increased. OPG, a decoy receptor for RANKL, blocks RANKL-RANK interaction and inhibits the activation of osteoclasts. The RANKL expression was outstandingly high in NC group, which may have resulted in increased bone resorption. The micro-CT results on the bone microstructure in the NC group corroborated this conclusion (Table 1). RANKL expression, on the other hand, was found to be significantly lower in the Sham, E, and TPEE-treated groups compared to the NC group. A dose-dependent upregulation in OPG gene expression was observed in TPEE-treated groups. This may be attributed to the bone remodeling mechanism in which osteoblasts express RANKL and OPG to regulate osteoclast differentiation. The ratio of OPG to RANKL in bone is a critical factor in regulating bone metabolism. Micro-CT results revealed that the Sham group has the highest BMD and BV/TV percent values, while all the treated groups did not differ significantly from the NC group (Table 1). In short, TPEE treatment showed inhibitory effect on bone resorption at the transcriptional level but not at the macroscopic level over the course of this treatment.

OCN is a small non-collagenous matrix protein found in bone that is produced during new cell synthesis and serves as a specific marker for bone formation and turnover [50]. Estrogen deficiency has been shown to increase serum OCN, CTX-1, and ALP levels, indicating increased bone turnover [51]. Interestingly, we discovered that T500 showed decreased serum OCN and CTX-1 levels with a significantly high phosphorus content in the serum among the groups. In osteoporotic women, calcium and phosphorus deficiencies reduce the formation of hydroxyapatite crystals, allowing free osteocalcin to circulate in the bloodstream. The increased phosphorus level in the T500 group may have contributed to the decrease in serum OCN levels. This suggests that treating the OVX rats with TPEE at a dose of 500 mg/kg could promote bone mineralization.

The OCN protein was derived from osteoblast cells and when combined with type 1 collagen forms an unmineralized flexible osteoid on which the osteoblasts reside. Type 1 collagen, which is also synthesized by osteoblasts, accounts for more than 90% of the protein in the bone matrix [52]. ColA gene encodes the α1 chain of type I collagen which is the major extracellular matrix component of bone [53]. The TPEE-treated groups produced significantly more ColA protein in bone than the Sham group, but no significant increase in tibial OCN protein. This finding suggests that OCN protein may be a limiting factor for successful bone mineralization. This is because type 1 collagen and OCN contributed to the mineralization of the bone matrix, but our results showed that the treated groups showed only a slight increase in BMD when compared to the NC group. According to another study, as BMD decreases, OCN activity and synthesis increase [10]. In general, the BMD of the TPEE-treated groups was 1.78-fold lower than that of the Sham group, but there were no significant differences in serum OCN levels, as determined by micro-CT analysis. Our study found no correlation between serum OCN levels and BMD.

When bone ER levels in tibia tissue homogenates were determined, it was discovered that the T250 and T500 groups had significantly higher levels than the NC group. The bone ER concentration in this case reflected the presence of all ERs (ERα and -β). A previous report indicated that the ER found in osteoclasts induces osteoclast apoptosis, resulting in decreased bone resorption [52]. The increased bone ER level in our study allowed us to infer that TPEE treatment increased the level of bone ER, which may contribute to the induction of osteoclast cell death. In the absence of endogenous estrogen, ER gene expression was significantly reduced in the NC group, but it was significantly upregulated in the T250 and T500 groups. ER levels in osteoblasts and mesenchymal cells decrease in women over the age of 40 [54]. Mesenchymal cells play critical roles in bone regeneration, particularly in bone differentiation and formation [55]. This corroborates the slower bone repairing processes in the older individuals. In OVX rats, TPEE maintains high levels of ER in the bone, possibly enhancing bone formation.

One of the etiologic factors for osteoporosis include kidney dysfunction, as recorded in the present study by the increased serum level of urea and CRE and decreased serum protein level, with increased urinary calcium loss, indicating defective calcium absorption mechanisms [56]. The levels of serum ALP, CRE and BUN obtained in this study showed no significant increment in the treated groups compared to the NC and Sham operated group. Biomarkers have demonstrated a high potential for being a useful, relatively inexpensive, and non-invasive tool for assessing osteoporosis. However, analyzing serum biomarkers in isolation may make it difficult to determine whether a subject is at risk of osteoporosis. A combination of serum biomarkers could be used as input features for osteoporosis prediction.

Although the ranks for CRE, TBIL, and ALP fluctuated throughout the course of the 50 iterations of the feature selection process, the median aggregate ranks for CRE and ALP were both 1. Therefore, only TBIL was eliminated from the input features. There has been no consensus regarding the relationship between TBIL and osteoporosis in previous studies. YJ Lee, JY Hong, SC Kim, JK Joo, YJ Na and KS Lee [57] reported a positive correlation of TBIL with femur BMD in postmenopausal Korean women. However, another study on postmenopausal Korean women without potential liver disease reported an independent and inverse association between TBIL and the prevalence of osteoporosis but a positive correlation of TBIL with BMD was observed [58]. Due to the fact that RFECV scored and ranked features by iterating through different combinations of features for a given total number of features, the fluctuation in the ranking of TBIL may imply that the association between TBIL and osteoporosis is not direct, and that other biomarkers may have acted as a mediator or moderator between TBIL and osteoporosis. To better understand this, future research may focus on the interactions and relationships between the biomarkers associated with osteoporosis severity.

The results in Table 2 indicate that the selected biomarkers performed reasonably well in predicting the severity of osteoporosis in the test subjects. This finding is significant because it demonstrates that using the biomarkers yielded from RFECV not only ensure the quality of the subsequent machine learning performance, but this method can also be applied in combination with BMD measurement by DXA to improve the accuracy of early osteoporosis assessment in the high-risk group. A higher average F1-score and a higher average accuracy over 50 iterations indicated that the model could predict more accurately, whereas lower standard deviation of F1-score and lower standard deviation of accuracy over the 50 iterations indicated that the model's prediction was more stable.

The standard deviation illustrated in Fig. 6 corresponds to the difference in performances of a model over 50 iterations of training and testing due to the non-deterministic nature of the 5 machine learning models used. In other words, different results may be obtained even if given the same model and the same dataset. The model’s non-deterministic nature is caused by the random initialization of the model’s parameters, where the initial parameter values will affect the final optimized parameter values. Therefore, 50 training–testing iterations were carried out for each of the 5 machine learning models to avoid biases derived from the non-deterministic nature of the models.

There are 3 reasons why the standard deviation illustrated in Fig. 6 is acceptable. Firstly, the perceived high standard deviation can be mitigated by ensemble learning such as aggregating (e.g., averaging) the 50 results obtained. For instance, a sample will be labelled as osteoporosis if 35 out of the 50 models predicts osteoporosis. Secondly, the common practice is only to use the best model for prediction. However, we insisted on presenting the standard deviation (difference in performance of the same model) over 50 repetitions because the results obtained can be bias if the model is only trained and tested once. This bias is caused by the non-deterministic nature of the 5 machine learning models used in this study. In other words, different results will be yielded even when the exact same data is used to train an exact same model. The inconsistent results over each repetition are caused by the random initialization of the model’s parameters before training and the initial values of a model’s parameters affects their final optimized values. Thirdly, a t-test results was presented (Table 3) to show that the outperformance of the XGBoost over other models is statistically significant despite the perceived high standard deviation. Hence, the perceived higher standard deviation is acceptable.

XGBoost was the best performing classification model based on performance metrics. In comparison to the other four models, it has the highest average F1-score and average accuracy while maintaining the lowest standard deviation of F1-score and standard deviation of accuracy over the 50 iterations. This is consistent with several other previous studies involving XGBoost [36, 59–61].

## Conclusions

In short, TPEE down-regulated the expression of RANKL, a gene involved in the initiation of osteoclast differentiation and bone resorption, and upregulated ERβ gene expression. Our findings indicated that TPEE inhibited bone resorption via inhibiting the RANKL pathway in OVX rats at the transcriptional level but not at the macroscopic level throughout the course of treatment in this study. Additionally, TPEE maintained the estrogen dependent characteristics of the uterus in OVX rats. Furthermore, our findings showed that TBIL was not an important feature for predicting bone quality as assessed by RFECV among the serum biomarkers tested. On top of that, RFECV combined with the XGBoost model was able to achieve the highest prediction accuracy among the other classification models, despite having a limited number of features. This combination of tools has the potential to be a powerful tool for rapidly assessing and diagnosing postmenopausal osteoporosis.

## Supplementary Information

**Additional file 1**. Chemical composition analysis of TPEE. The representative UPLC chromatogram (A) and MS spectra (C) of TPEE. The retention time and the m/z ratio (B) of the indicative compounds (daidzein, genistein, formononetin and biochanin A) found in TPEE.

**Additional file 2**. Conversion of TPEE doses and functional indicator components, formononetin and biochanin A, in rats to human equivalent doses based on body surface area.

**Additional file 3**. The optimum PCR condition and the sequences of the primers used for in vivo analysis.

**Additional file 4**. Primary and secondary biomarkers collected from the biochemistry analysis and enzyme-linked immunosorbent assay (ELISA).

**Additional file 5**. Classification of severity of osteoporosis measured by T-score of Bone Mass Density (BMD).

**Additional file 6**. The distribution of samples according to the severity groups.

**Additional file 7**. Modelling framework.

**Additional file 8**. Typical chromatograms of biochanin A, formononetin and TPEE for specificity analysis.

**Additional file 9**. Accuracy and precision of the high-performance liquid chromatography (HPLC) assay for biochanin A (BCA) and formononetin (FMT).

**Additional file 10**. LOD and LOQ results for biochanin A (BCA) and formononetin (FMT).

**Additional file 11**. Linearity of the HPLC method for analysis of biochanin A (BCA) and formononetin (FMT).

**Additional file 12**. The levels of ER protein in OVX rats’ tibia determined by enzyme-linked immunosorbent assay. Data are expressed as mean ± SD (n = 6). Data were presented as mean ± SD, n=6. *p <0.05; **p < 0.01; ***p < 0.001; ****p < 0.0001 determined by two-way ANOVA test, followed by Tukey’s post hoc test.

**Additional file 13**. Selected serum biochemical marker levels in control and different treated groups. Data are mean ± SD of 6 rats in each group. Significance at P<0.05. aSignificant difference between OVX treated (P, E, T125, T250, T500) groups and OVX negative control group (NC). bSignificant difference compared with Sham operated group (Sham).

## Data Availability

The typical programming script for feature ranking and modeling was deposited and can be access with the https://doi.org/10.5281/zenodo.6374504. The datasets used and/or analyzed during the current study are available from the corresponding author on reasonable request.

## Abbreviations

ALB: Serum albumin
ALP: Alkaline phosphatase
ALT: Serum alanine aminotransferase
ANN: Artificial Neural Network
ANOVA: Analysis of variance
AST: Serum aspartate aminotransferase
BCA: Biochanin A
BMD: Bone mineral density
BMI: Body mass index
BUN: Blood urea nitrogen
BV/TV: Percentage of bone volume/total volume
CE: Cross-entropy
ColA: Collagen type 1 (α)
CRE: Serum creatinine
CTX-1: Serum C-telopeptide of type I collagen
DT: Decision Tree
DXA or DEXA: Dual-energy x-ray absorptiometry
DZN: Daidzein
E: Estradiol
ELISA: Enzyme-linked immunosorbent assay
ER: Estrogen receptor
FMT: Formononetin
GMP: Good Manufacturing Practice
GNT: Genistein
H&E: Hematoxylin and eosin
HPLC: High performance liquid chromatography
LOD: Limit of detection
LOQ: Limit of quantification
micro-CT: Micro-Computed Tomography
NC: Negative control
OCN: Osteocalcin
OPG: Osteoprotegerin
OVX: Ovariectomized
PomE: Pomegranate extract
PTH: Parathyroid hormone
RANKL: Nuclear factor-kappa B ligand
RF: Random Forest
RFECV: Recursive Feature Elimination Cross Validation
RSD: Relative Standard Deviation
RT-PCR: Reverse-transcription Polymerase Chain Reaction
SD: Standard Deviation of the Response
SVM: Support-Vector Machine
Tb.N: Trabecular number
Tb.Sp: Trabecular spacing
Tb.Th: Trabecular thickness
TBIL: Serum total bilirubin
TPEE: *Trifolium pratense* Ethanolic extract
UPLC-ESI–MS/MS: Ultra-performance liquid chromatography-electrospray tandem mass spectrometry
