# Supplementary figures and images for: Serum biomarker-based osteoporosis risk prediction and the systemic effects of Trifolium pratense ethanolic extract in a postmenopausal model
Source: Chin Med. 2022 Jun 14;17:70. doi: 10.1186/s13020-022-00622-7 (PMC9199188; doi:10.1186/s13020-022-00622-7)

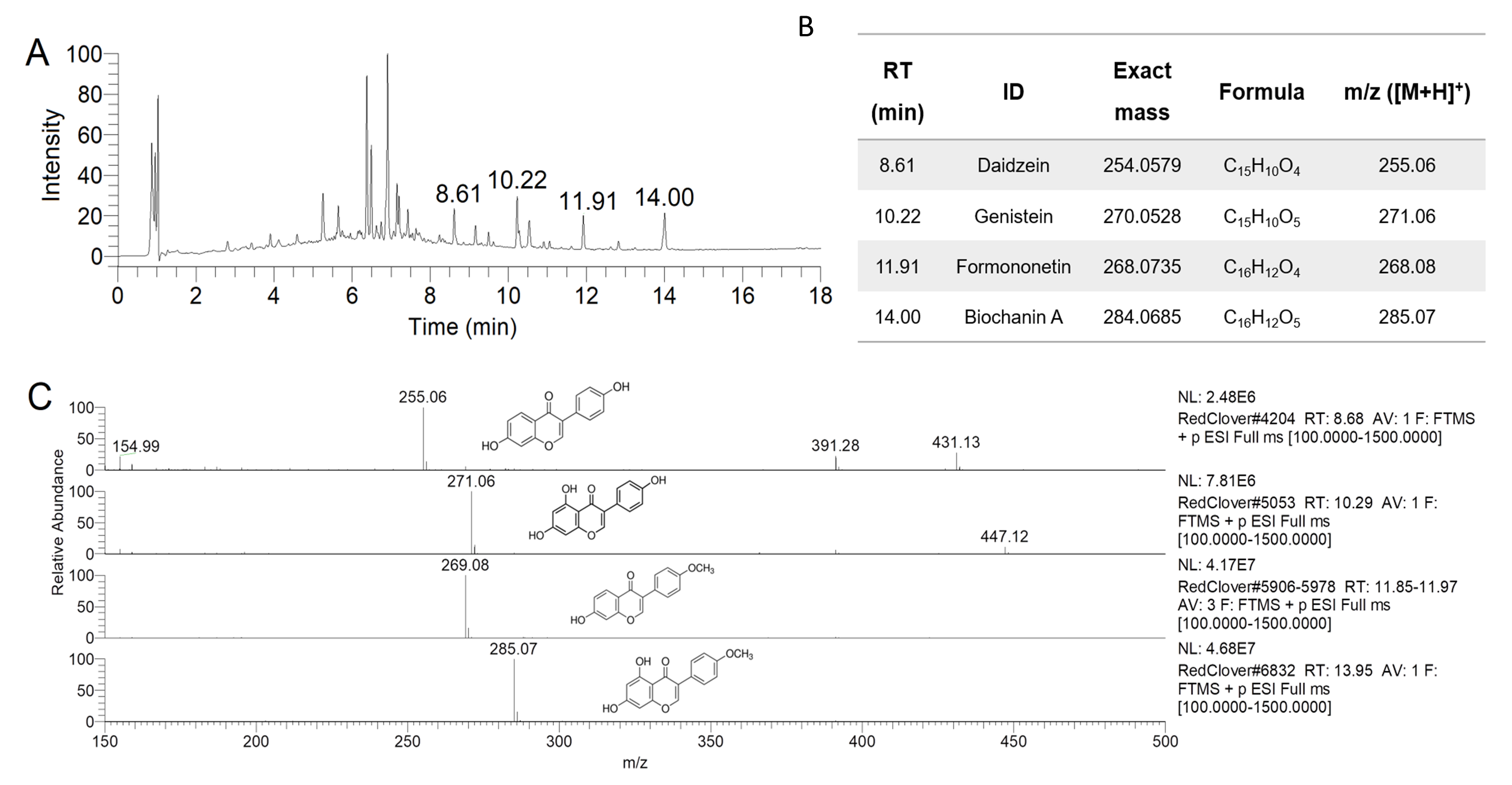

Supplement: Supplementary file 1 — Additional file 1. Chemical composition analysis of TPEE. The representative UPLC chromatogram (A) and MS spectra (C) of TPEE. The retention time and the m/z ratio (B) of the indicative compounds (daidzein, genistein, formononetin and biochanin A) found in TPEE. [file 13020_2022_622_MOESM1_ESM.tif]

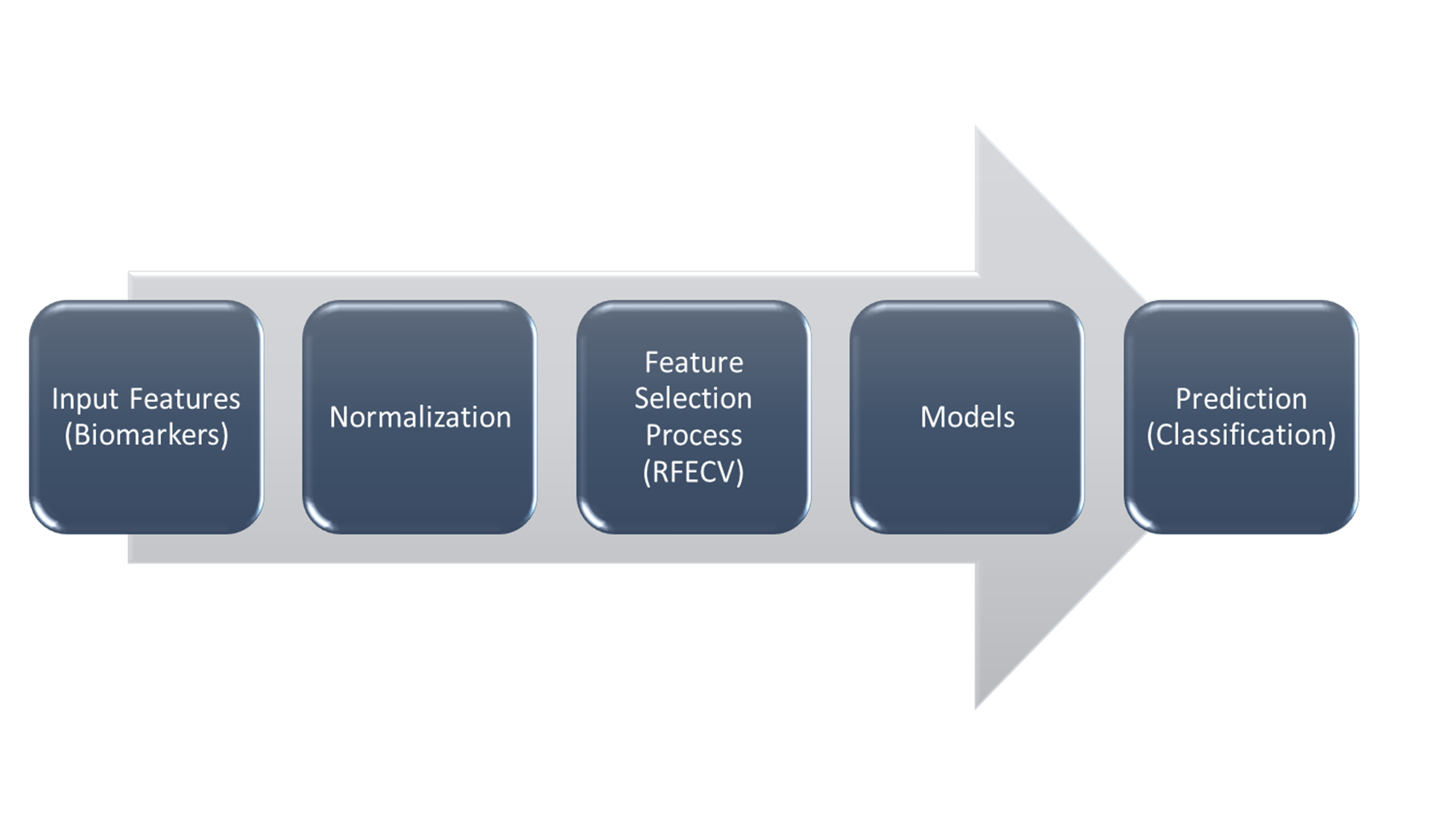

Supplement: Supplementary file 7 — Additional file 7. Modelling framework. [file 13020_2022_622_MOESM7_ESM.tif]

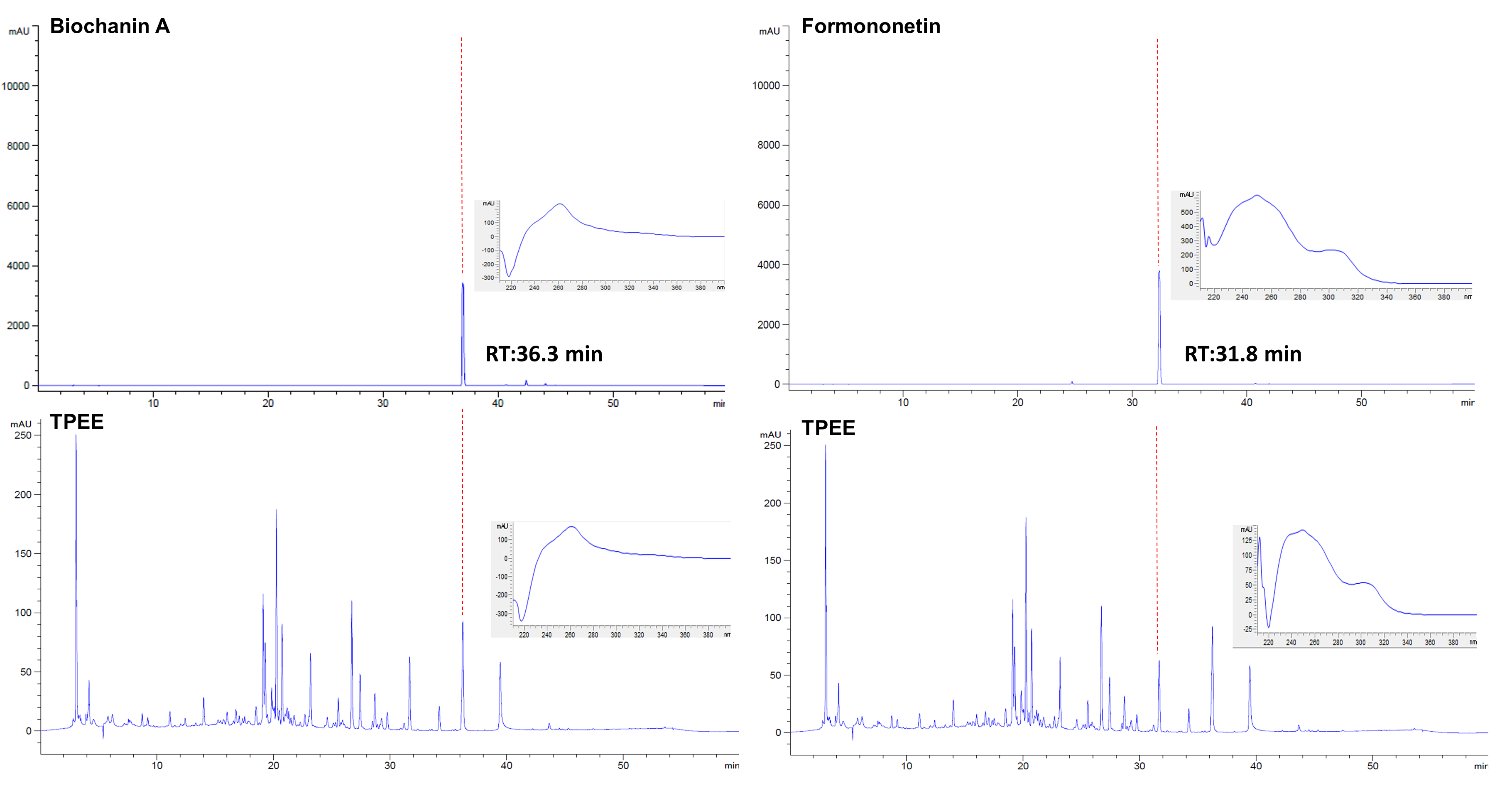

Supplement: Supplementary file 8 — Additional file 8. Typical chromatograms of biochanin A, formononetin and TPEE for specificity analysis. [file 13020_2022_622_MOESM8_ESM.tif]

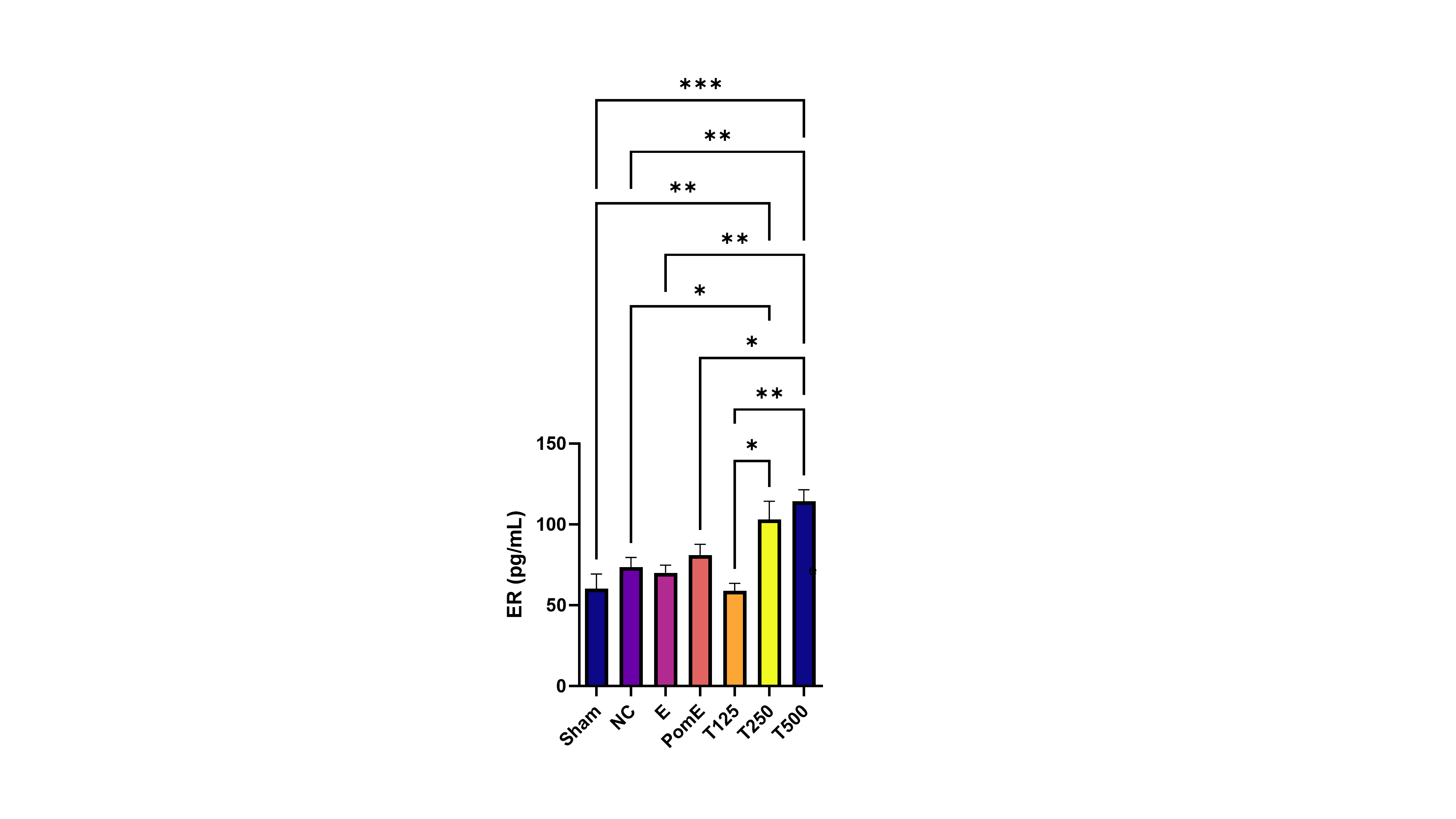

Supplement: Supplementary file 12 — Additional file 12. The levels of ER protein in OVX rats’ tibia determined by enzyme-linked immunosorbent assay. Data are expressed as mean ± SD (n = 6). Data were presented as mean ± SD, n=6. *p <0.05; **p < 0.01; ***p < 0.001; ****p < 0.0001 determined by two-way ANOVA test, followed by Tukey’s post hoc test. [file 13020_2022_622_MOESM12_ESM.tif]
