# Supplementary material for: Serum biomarker-based osteoporosis risk prediction and the systemic effects of Trifolium pratense ethanolic extract in a postmenopausal model
Source: Chin Med. 2022 Jun 14;17:70. doi: 10.1186/s13020-022-00622-7 (PMC9199188; doi:10.1186/s13020-022-00622-7)
Supplement: Supplementary file 2 — Additional file 2. Conversion of TPEE doses and functional indicator components, formononetin and biochanin A, in rats to human equivalent doses based on body surface area. [file 13020_2022_622_MOESM2_ESM.docx]

**Additional file 2.** Conversion of TPEE doses and functional indicator components, formononetin and biochanin A, in rats to human equivalent doses based on body surface area.

| TPEE dose administered to rat (mg/kg) | TPEE dose in human (g/kg) | Factor | TPEE dose in 60 kg human (g/60kg) | Formononetin (mg/60kg) | Biochanin A (mg/60kg) |
| --- | --- | --- | --- | --- | --- |
| 500 | 0.08 | 0.16 | 4.8 | 13.00 | 13.92 |
| 250 | 0.04 | 0.16 | 2.4 | 6.50 | 6.96 |
| 125 | 0.02 | 0.16 | 1.2 | 3.25 | 3.48 |
