# Supplementary material for: Serum biomarker-based osteoporosis risk prediction and the systemic effects of Trifolium pratense ethanolic extract in a postmenopausal model
Source: Chin Med. 2022 Jun 14;17:70. doi: 10.1186/s13020-022-00622-7 (PMC9199188; doi:10.1186/s13020-022-00622-7)
Supplement: Supplementary file 3 — Additional file 3. The optimum PCR condition and the sequences of the primers used for in vivo analysis. [file 13020_2022_622_MOESM3_ESM.docx]

**Additional file 3.** The optimum PCR condition and the sequences of the primers used for *in vivo* analysis.

| **Gene** | **Forward** | **Reverse** |
| --- | --- | --- |
| **RANKL** | CCAGCATCAAAATCCCAAGT | TGAAAGCCCCAAAGTACGTC |
| **OPG** | CACTGCACAGTCAGGAGGAA | TTCCTCACATTCGCACACTC |
| **OCN** | GCACATGACCCCCAATTAGT | CAGAGAGAGGGTCCTCATGG |
| **ALP** | ACGTCAATTAACGGCTGACA | AGTCCTACCTGGCACAAATGA |
| **ColA** | TTCATGACCACTGCCTGTTC | AAGTCACGAGGCCTTACCTG |
| **ER α** | CCGAACGGATTCTACCAAAA | AGAAGAATGTCGCCCAGAGA |
| **ER β** | GAAGCTGAACCACCCAATGT | CAATCATGTGCACCAGTTCC |
