# Supplementary material for: Serum biomarker-based osteoporosis risk prediction and the systemic effects of Trifolium pratense ethanolic extract in a postmenopausal model
Source: Chin Med. 2022 Jun 14;17:70. doi: 10.1186/s13020-022-00622-7 (PMC9199188; doi:10.1186/s13020-022-00622-7)
Supplement: Supplementary file 4 — Additional file 4. Primary and secondary biomarkers collected from the biochemistry analysis and enzyme-linked immunosorbent assay (ELISA). [file 13020_2022_622_MOESM4_ESM.docx]

**Additional file 4.** Primary and secondary biomarkers collected from the biochemistry analysis and enzyme-linked immunosorbent assay (ELISA)

| **Primary** | **Secondary** |
| --- | --- |
| Alkaline phosphatase (ALP)  Calcium (Ca)  Phosphorous (PHOS)  C-terminal telopeptide (CTX-1)  Deoxypyridinoline (DPD)  Osteocalcin (OCN)  Estrogen (E2)  Creatinine (CRE) | Aspartate transaminase (AST)  Alanine transaminase (ALT)  Total protein (TP)  Albumin (ALB)  Blood urea nitrogen (BUN)  Glucose (GLU)  Triglyceride (TG)  Total bilirubin (TBIL) |
