# Supplementary material for: Serum biomarker-based osteoporosis risk prediction and the systemic effects of Trifolium pratense ethanolic extract in a postmenopausal model
Source: Chin Med. 2022 Jun 14;17:70. doi: 10.1186/s13020-022-00622-7 (PMC9199188; doi:10.1186/s13020-022-00622-7)
Supplement: Supplementary file 5 — Additional file 5. Classification of severity of osteoporosis measured by T-score of Bone Mass Density (BMD). [file 13020_2022_622_MOESM5_ESM.docx]

**Additional file 5.** Classification of severity of osteoporosis measured by T-score of Bone Mass Density (BMD).

| **T-Score, T** | **Severity Group** |
| --- | --- |
| T >= - 1 | No Osteoporosis |
| -1 < T <= -2.5 | Low Bone Density (Osteopenia) |
| -2.5 >= T | Osteoporosis |
