# Supplementary material for: Serum biomarker-based osteoporosis risk prediction and the systemic effects of Trifolium pratense ethanolic extract in a postmenopausal model
Source: Chin Med. 2022 Jun 14;17:70. doi: 10.1186/s13020-022-00622-7 (PMC9199188; doi:10.1186/s13020-022-00622-7)
Supplement: Supplementary file 6 — Additional file 6. The distribution of samples according to the severity groups. [file 13020_2022_622_MOESM6_ESM.docx]

**Additional file 6.** The distribution of samples according to the severity groups.

| **Severity Group** | **No Osteoporosis** | **Osteopenia** | **Osteoporosis** |
| --- | --- | --- | --- |
| **Distribution** | 24.66% | 2.74% | 72.6% |
