# Supplementary material for: Serum biomarker-based osteoporosis risk prediction and the systemic effects of Trifolium pratense ethanolic extract in a postmenopausal model
Source: Chin Med. 2022 Jun 14;17:70. doi: 10.1186/s13020-022-00622-7 (PMC9199188; doi:10.1186/s13020-022-00622-7)
Supplement: Supplementary file 9 — Additional file 9. Accuracy and precision of the high-performance liquid chromatography (HPLC) assay for biochanin A (BCA) and formononetin (FMT). [file 13020_2022_622_MOESM9_ESM.docx]

**Additional file 9.** Accuracy and precision of the high-performance liquid chromatography (HPLC) assay for biochanin A (BCA) and formononetin (FMT).

| Accuracy | Parameters | Spiked analyte (mg) | | | | | | | | | | |  |  |
| --- | --- | --- | --- | --- | --- | --- | --- | --- | --- | --- | --- | --- | --- | --- |
|  |  | **0.015625** | | | **0.03125** | | | **0.0625** | | | **0.125** | |  |  |
| BCA | Average recovery (%) | 91.27 ± 0.76 | | | 89.85 ± 2.60 | | | 84.58 ± 1.82 | | | 87.84 ± 1.61 | |  |  |
|  | Total average recovery (%) | 88.38 | | | | | | | | | | |  |  |
|  | Recovery range (%) | 84.58-91.27 | | | | | | | | | | |  |  |
| FMT | Average recovery (%) | 100.28 ± 0.71 | | | 100.22 ± 0.20 | | | 99.63 ± 1.33 | | | 106.36 ± 0.15 | |  |  |
|  | Total average recovery (%) | 101.63 | | | | | | | | | | |  |  |
|  | Recovery range (%) | 99.63-106.36 | | | | | | | | | | |  |  |
| Precision (Repeatability) | **Parameters** | | | **Contents (mg)** | | | | | | | | | |  |
|  |  |  |  | **15** | | **20** | | | **25** | | | **30** | |  |
| BCA | Concentration analyzed (mg/g) | | | 1.02 ± 0.01 | | 1.00 ± 0.01 | | | 1.03 ± 0.01 | | | 1.03 ± 0.01 | |  |
|  | RSD (%) | | | 0.58 | | 0.68 | | | 0.71 | | | 1.09 | |  |
|  | RSD range (%) | | | 0.58-1.09 | | | | | | | | | |  |
| FMT | Concentration analyzed (mg/g) | | | 0.87 ± 0.00 | | 0.86 ± 0.00 | | | 0.87 ± 0.00 | | | 0.86 ± 0.01 | |  |
|  | RSD (%) | | | 1.02 | | 0.88 | | | 0.37 | | | 1.03 | |  |
|  | RSD range (%) | | | 0.37-1.03 | | | | | | | | | |  |
| Precision (Reproducibility) | **Parameters** | | **Laboratory** | | | | | | | | | | | |
|  |  |  | **A** | | | | **B** | | | **C** | | | | |
| BCA | Concentration analyzed (mg/g) | | 1.02 ± 0.02 | | | | 1.02 ± 0.01 | | | 1.02 ± 0.01 | | | | |
|  | RSD (%) | | 1.61 | | | | 0.88 | | | 1.11 | | | | |
| FMT | Concentration analyzed (mg/g) | | 0.87 ± 0.01 | | | | 0.87 ± 0.01 | | | 0.86 ± 0.01 | | | | |
|  | RSD (%) | | 1.26 | | | | 0.96 | | | 1.32 | | | | |
