# Supplementary material for: Serum biomarker-based osteoporosis risk prediction and the systemic effects of Trifolium pratense ethanolic extract in a postmenopausal model
Source: Chin Med. 2022 Jun 14;17:70. doi: 10.1186/s13020-022-00622-7 (PMC9199188; doi:10.1186/s13020-022-00622-7)
Supplement: Supplementary file 10 — Additional file 10. LOD and LOQ results for biochanin A (BCA) and formononetin (FMT). [file 13020_2022_622_MOESM10_ESM.docx]

**Additional file 10.** LOD and LOQ results for biochanin A (BCA) and formononetin (FMT).

|  | Parameters | R1 | R2 | R3 |
| --- | --- | --- | --- | --- |
| BCA | Gradient | 7111.2 | 7329.4 | 7354.7 |
|  | Average gradient | 7265.0 | | |
|  | y-intercept | 122.27 | 111.61 | 94.39 |
|  | y-intercept standard deviation (σ) | 14.068 | | |
|  | LOQ | 0.01936 | | |
|  | LOD | 0.00639 | | |
| FMT | Gradient | 4898.8 | 5057.1 | 5050.9 |
|  | Average gradient | 5002.2 | | |
|  | y-intercept | 67.25 | 56.72 | 51.86 |
|  | y-intercept standard deviation (σ) | 7.868 | | |
|  | LOQ | 0.01573 | | |
|  | LOD | 0.00519 | | |
