# Supplementary material for: Serum biomarker-based osteoporosis risk prediction and the systemic effects of Trifolium pratense ethanolic extract in a postmenopausal model
Source: Chin Med. 2022 Jun 14;17:70. doi: 10.1186/s13020-022-00622-7 (PMC9199188; doi:10.1186/s13020-022-00622-7)
Supplement: Supplementary file 11 — Additional file 11. Linearity of the HPLC method for analysis of biochanin A (BCA) and formononetin (FMT). [file 13020_2022_622_MOESM11_ESM.docx]

**Additional file 11.** Linearity of the HPLC method for analysis of biochanin A (BCA) and formononetin (FMT).

| Linearity | BCA | FMT |
| --- | --- | --- |
| Concentration (mg/ml) | Peak area (Au) | Peak area (Au) |
| 0.125 | 9167.0 | 6297.8 |
| 0.0625 | 4691.7 | 3208.0 |
| 0.03125 | 2401.6 | 1635.8 |
| 0.015625 | 1247.2 | 843.2 |
| 0.0078125 | 635.1 | 423.2 |
| Gradient (a) | 7265.0 | 5002.2 |
| y-intercept (b) | 109.44 | 58.611 |
| Coefficient of determination (r^2^) | 0.9999 | 0.9999 |
