# Supplementary material for: Serum biomarker-based osteoporosis risk prediction and the systemic effects of Trifolium pratense ethanolic extract in a postmenopausal model
Source: Chin Med. 2022 Jun 14;17:70. doi: 10.1186/s13020-022-00622-7 (PMC9199188; doi:10.1186/s13020-022-00622-7)
Supplement: Supplementary file 13 — Additional file 13. Selected serum biochemical marker levels in control and different treated groups. Data are mean ± SD of 6 rats in each group. Significance at P<0.05. aSignificant difference between OVX treated (P, E, T125, T250, T500) groups and OVX negative control group (NC). bSignificant difference compared with Sham operated group (Sham). [file 13020_2022_622_MOESM13_ESM.docx]

**Additional file 13.** Selected serum biochemical marker levels in control and different treated groups.

|  | TP (g/dL) | CRE (mg/dL) | ALP (U/L) | TG (mg/dL) | Ca (mg/dL) | Phos (mg/dL) | BUN (mg/dL) |
| --- | --- | --- | --- | --- | --- | --- | --- |
| NC | 6.47 ± 0.23 | 0.60 ± 0.09 | 246.83 ± 26.13 | 38.33 ± 2.42 | 9.90 ± 0.19 | 6.27 ± 0.65 | 24.73 ± 2.74 |
| Sham | 6.15 ± 0.29 | 0.65 ± 0.08 | 246.17 ± 45.28 | 26.00 ± 8.58^a^ | 10.12 ± 0.21 | 6.93 ± 0.15 | 23.90 ± 2.90 |
| E | 5.88 ± 0.12^b^ | 0.60 ± 0.01 | 222.50 ± 72.37 | 29.83 ± 6.91 | 9.87 ± 0.12 | 6.50 ± 0.67 | 16.00 ± 0.89^a^ |
| PomE | 5.65 ± 0.19 | 0.63 ± 0.05 | 227.00 ± 37.11 | 26.83 ± 6.01^a^ | 9.80 ± 0.13 | 7.22 ± 0.76 | 24.18 ± 3.75 |
| T125 | 5.73 ± 0.05^ab^ | 0.60 ± 0.01 | 235.33 ± 40.25 | 25.67 ± 2.16^a^ | 9.80 ± 0.09^b^ | 7.42 ± 0.22 | 24.48 ± 0.80 |
| T250 | 5.67 ± 0.18^ab^ | 0.67 ± 0.05 | 278.67 ± 16.57 | 23.33 ± 3.72^a^ | 9.77 ± 0.19^b^ | 6.90 ± 0.58 | 21.53 ± 0.45 |
| T500 | 5.88 ± 0.10^a^ | 0.65 ± 0.05 | 290.50 ± 42.04 | 23.67 ± 5.39^a^ | 9.72 ± 0.19^b^ | 8.65 ± 0.50^b^ | 24.67 ± 1.77 |

Data are mean ± SD of 6 rats in each group. Significance at P<0.05.

^a^Significant difference between OVX treated (P, E, T1, T2, T3) groups and OVX negative control group (NC). ^b^Significant difference compared with Sham operated group (Sham).
